# Micro-Executor of Natural Products in Metabolic Diseases

**DOI:** 10.3390/molecules28176202

**Published:** 2023-08-23

**Authors:** Jinxin Liu, Huanwen Chen, Xiaoli Li, Chunmei Song, Li Wang, Deguo Wang

**Affiliations:** 1 Food and Pharmacy College, Xuchang University, Xuchang 461000, China; liujinxin2013@163.com (J.L.); zhiya007@163.com (C.S.); 2 Center for Agricultural and Rural Development, Zhangdian District, Zibo 255000, China; zdqnab@163.com; 3 Zibo Digital Agriculture and Rural Development Center, Zibo 255000, China; zblixl@126.com; 4 School of Food Science and Technology, Jiangnan University, 1800 Lihu Avenue, Wuxi 214122, China; 5 Key Laboratory of Biomarker Based Rapid-Detection Technology for Food Safety of Henan Province, Xuchang University, Xuchang 461000, China

**Keywords:** natural products, miRNAs, glyco-lipid metabolism disorders, cardiovascular diseases

## Abstract

Obesity, diabetes, and cardiovascular diseases are the major chronic metabolic diseases that threaten human health. In order to combat these epidemics, there remains a desperate need for effective, safe, and easily available therapeutic strategies. Recently, the development of natural product research has provided new methods and options for these diseases. Numerous studies have demonstrated that microRNAs (miRNAs) are key regulators of metabolic diseases, and natural products can improve lipid and glucose metabolism disorders and cardiovascular diseases by regulating the expression of miRNAs. In this review, we present the recent advances involving the associations between miRNAs and natural products and the current evidence showing the positive effects of miRNAs for natural product treatment in metabolic diseases. We also encourage further research to address the relationship between miRNAs and natural products under physiological and pathological conditions, thus leading to stronger support for drug development from natural products in the future.

## 1. Introduction

Metabolic diseases, which encompass a variety of risk factors highly associated with obesity, diabetes, and cardiovascular diseases, have come to be regarded as public health challenges [1,2,3,4]. Due to their complex mechanisms of action, effective comprehensive treatments are still lacking. Even worse, the side effects of some curative drugs have been a major concern for their therapeutic usage [5,6,7]. Therefore, it is imperative to provide an effective treatment approach to overcome the aforementioned diseases.

Natural products that are extracted from the source and from concentrated, fractionated, and purified yielding, which are generally defined as bioactive compounds [8,9], have the ability to modulate lipid metabolism, improve insulin signaling, and protect against cardiovascular damage [10,11]. More importantly, natural products are widely distributed and readily available in nature [12]. To date, extensive studies have shown that plentiful drugs are derived from structural modification based on natural products [13]. MicroRNAs (miRNAs) and small noncoding RNAs are characterized by binding to the regulatory sites of 3′UTR of target mRNA, resulting in the inhibition of transcription or the promotion of degradation, accompanied by decreased protein synthesis [14,15]. Natural products could also ameliorate metabolic diseases by targeting abundant miRNAs [16,17,18]. Thus, the possibility for natural products to modify the abnormal patterns of these diseases is, at least in part, possible through a newly defined mechanism: the miRNAs cascade.

In this review, we summarize the positive effects of natural products on lipid and glucose metabolism disorders and cardiovascular diseases, explain the underlying molecular mechanisms, and provide the theoretical basis for metabolic diseases. We also highlight the regulatory effect of natural products on miRNAs in this therapeutic process. In the hope of the preferable utilization of existing data, we thus provide a new route for future drug discovery.

## 2. Research Methodology

A systematic search of the literature was performed in the PubMed and Web of Science databases (up to December 2022). The following text terms were used to identify any study candidates: (“microRNA(s)” OR “miRNA(s)” OR “miR”) AND (“natural product(s)” OR “plant(s)” OR “Herb(s)” OR “extract(s)”) AND (“metabolic disease(s)” OR “glyco-lipid metabolism disorders” OR “lipid metabolism disorder(s)” OR “Glucose metabolism disorder(s)” OR “cardiovascular disease(s)” OR “obesity” OR “diabetes” OR “glucose” OR “lipid” OR “fat” OR “adipocyte” OR “metabolism”). Moreover, we hand-searched the citation lists of the included studies to identify the relevant literature. As for data extraction, the titles, keywords, abstracts, and full texts of all enrolled articles were screened. Any duplicates and irrelevant studies were excluded; traditional Chinese medicine formulas, extracts, or combinations with unclear functional components were also excluded from the literature. The useful data were extracted from the relevant qualified literature into specifically designed spreadsheets. The following data were included: the first author, country, year of publication, natural products, functional components, experimental models, dosage and duration of treatment, observed effects, associated miRNAs, and target genes.

## 3. Effects of Natural Products on Lipid Metabolism Disorders

Lipid metabolism is a crucial and complex biochemical reaction in the body, and diseases caused by lipid metabolism disorders are common in modern society, such as obesity and hyperlipidemia [19]. Lipids are known to be important substances in energy storage and energy supply. Hence, the proper amount of adipose tissue is necessary for the human body. In general, however, patients have difficulty sticking to a long-term diet and physical activity regimen to combat these metabolic disorders. Therefore, food components that ameliorate the risk factors associated with these diseases can facilitate dietary-based therapies [16]. Dietary natural products have long been of great interest for improving lipid metabolism by modulating miRNA expression.

### 3.1. Regulatory Effects on Fatty Acid Synthesis and Decomposition

It is well known that fatty acids are the simplest type of lipids and are the building blocks of many more complex fats. Furthermore, they are also perceived as one of the main sources of energy on account of releasing a lot of energy during oxidation into CO_2_ and H_2_O in the case of a sufficient oxygen supply. Therefore, the role of fatty acids in the processes of lipogenesis and lipodieresis cannot be ignored. Recently, investigators have examined the regulatory effects of natural products on lipogenesis and lipodieresis, resulting in improved lipid metabolism through the management of diverse miRNAs (Table 1 and Table 2).

Specifically, miR-122 and miR-33 are two of the best-studied miRNAs involved in the regulation of lipid metabolism [53]. As is shown in Table 1 and Table 2, numerous pieces of evidence have revealed that grape seed proanthocyanidin extract treatments reduced fatty acid synthesis and de novo lipogenesis, increased liver cholesterol efflux to high-density lipoprotein (HDL) formation by decreasing the expression of miR-122 and miR-33, which could regulate several genes that control fatty acid and transcriptional regulatory factors, such as fatty acid synthase (FAS) and peroxisome proliferator-activated receptor beta/delta (PPARβ/δ), as well as genes that regulate fatty acid β-oxidation, such as ATP-binding cassette transporter A1 (ABCA1) and carnitine palmitoyltransferase 1a (CPT1a), respectively [16,25,26,27,29,54]. Further detection revealed that the levels of total cholesterol (TC), triglyceride (TG), and low-density lipoprotein cholesterol (LDL-C) were reduced while the level of high-density lipoproteins cholesterol (HDL-C) was enhanced in a dose-dependent manner [16,25,26,27]. *Averrhoa carambola*-free phenolic extract, citrus peel flavonoids, lychee pulp phenolics, mulberry fruit extract, and portulaca oleracea extract treatments could also improve lipid metabolism in in vitro and in vivo studies; the underlying mechanism was miR-33 or miR-122-mediated changes in the signaling pathways [20,22,33,34,37,38]. However, the opposite expression of miR-122 was reflected in a natural product experiment using coffee polyphenols, which could enhance energy metabolism and reduce lipogenesis by targeting sterol regulatory element binding protein (SREBP) 1c mediated by miR-122 [23]. Accountably, SREBP1c, one of the three isoforms of SREBPs, comes into play in fatty acid synthesis and metabolism [55,56]. It potentially illustrates the point that the same type of miRNAs can act on a variety of target genes with different expressions. Similarly, the same target gene may also be regulated by multiple miRNAs. Recent studies have shown that miR-103 and miR-107 reduced obesogenic diet-induced hepatic steatosis via decreasing the protein expression of SREBP1 in resveratrol-treated rats [49], and pseudoprotodioscin promoted cholesterol effluxion through targeting SREBP1c and SREBP2 mediated by miR-33a/b in an in vitro experiment [48]. In addition, distinctively, the overexpression of hepatic miR-98 induced by oleanolic acid, an active component of the traditional Chinese herb olea europaea, increased the degradation of peroxisome proliferator-activated receptor gamma coactivator-1beta (PGC1β), known as a transcriptional co-activator of SREBP-1 and the master regulator of hepatic lipogenesis [46,57].

Intuitively, both FAS and SREBP1 are involved in the process of fatty acid synthesis, and the connection between them is found in the following experiments. SP1 transcription factor (SP1), an important member of the ubiquitously expressed SP/KLF transcription factor family, acts together with SREBP1 to synergistically activate the promoter of the FAS gene and is involved in de novo lipogenesis [58]. Hence, resveratrol reduced the expression of SP1 through upregulating miR-539, along with decreasing the expression of the SREBP1 protein and FAS gene in vivo and in vitro [50]. Nevertheless, the correlation between them still needs to be systematically and intensively investigated beyond all doubt.

Lipid metabolism is a complex process, and natural products can regulate lipogenesis and lipodieresis in a variety of ways. Zerumbone is a cyclic sesquiterpene isolated from the wild ginger *Zingiber zerumbet smith*. It has been proved that zerumbone could improve lipid metabolism disorder by reducing lipogenesis and increasing fatty acid oxidation [52]. For one thing, zerumbone acted as a miR-146b inhibitor and downregulated miR-146b, leading to the activation of sirtuin type 1 (Sirt1), which induced the de-acetylation of forkhead box O1 (FOXO1) and peroxisome proliferator-activated receptor gamma coactivator-1alpha (PGC1α); for another, zerumbone induced the phosphorylation of AMP-activated protein kinase (AMPK), which could limit fatty acid efflux from adipocytes and favor fatty acid oxidation, as well as decrease de novo fatty acid synthesis through the phosphorylation-mediated inhibition of acetyl-CoA carboxylase (ACC) [59,60,61] and also activated Sirt1 indirectly [52]. With these similar natural product experiments, miR-27a/b, miR-96, miR-34a, miR-194, and miR-355 also participated in the process of lipid metabolism by targeting Sirt1 or FOXO1, which could both increase energy expenditure and the clearance of lipid accumulation [21,28,30,31,35,36]. And ginger extract could enhance AMPK activity and ameliorate obesity and inflammation by regulating miRNAs expressions in high-fat diet (HFD)-fed rats [24].

**Figure 1:**
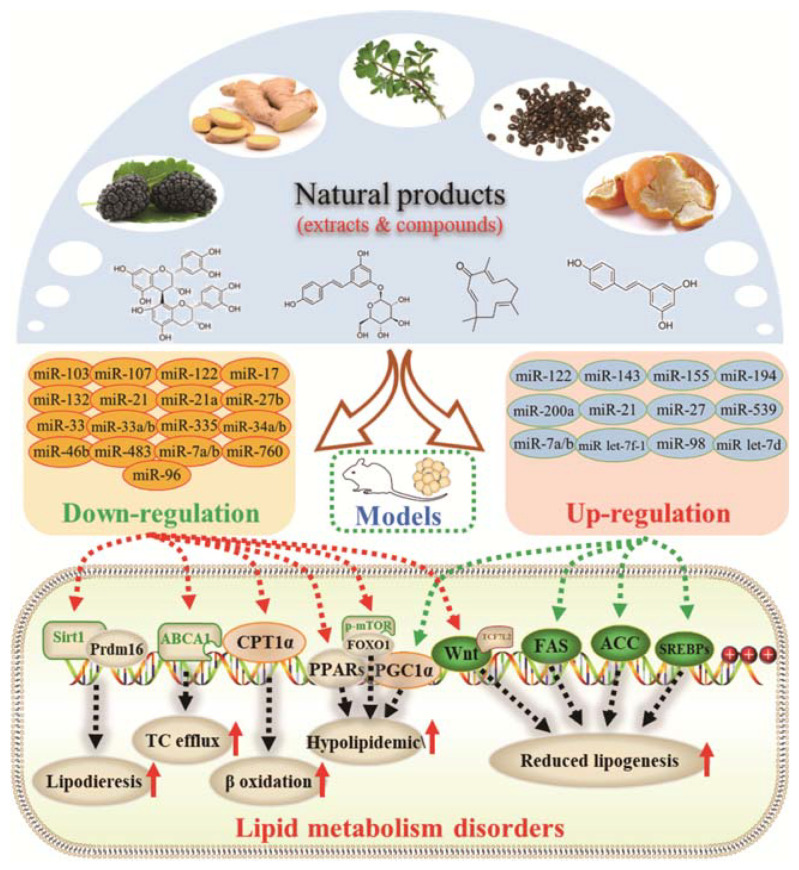
Schematic illustration of the main mechanisms by which natural products improve lipid metabolism disorders mediated by miRNAs. The red arrow means an increase, and the green arrow means a decrease.

Based on the present studies, regulating miRNAs is potentially becoming a dominant feature in terms of natural products regulating lipid metabolism (Figure 1). On the one hand, it can inhibit fatty synthesis by reducing fatty acid synthesis and increasing fatty acids mobilization. On the other hand, it can also accelerate lipodieresis by enhancing the oxidation and phosphorylation of fatty acids.

### 3.2. Inhibitory Effects on Adipocyte Differentiation and Accumulation

From the perspective of the cellular level, however, the growth of adipose tissue is the result of an increase in the number of adipocytes and the volume of individual cells [51]. The former contributes to promoting pre-adipocyte differentiation into mature adipocytes, whereas the latter is due to lipid accumulation. Here, we summarized the functional role of natural products in this regard, as well as their potential mechanisms of action (Figure 1 and Table 1 and Table 2).

Adipocyte differentiation is a highly precisely regulated cellular process. Ahead of terminal differentiation, the mitotic clonal expansion (MCE) of stimulated pre-adipocytes is an essential procedure in adipocyte differentiation. Moreover, the transcriptional activation of adipocyte-specific functional genes is closely related to their differentiation [62]. 3T3-L1 pre-adipocytes have long been considered as the “gold standard” for investigating pre-adipocyte differentiation in vitro [63,64]. There has been evidence that the MCE process could be delayed by persimmon tannin by enhancing the expression of miR-27 in 3T3-L1 pre-adipocytes [47]. Furthermore, multiple transcriptional factors, including peroxisome proliferator-activated receptor-gamma (PPARγ) and CCAAT/enhancer-binding protein alpha (C/EBPα) were also attenuated by miR-27, resulting in a decrease in adipocyte-specific genes, such as adipocyte fatty acid binding protein (aP2) and lipoprotein lipase (LPL). Similarly, the MCE process was blocked by miR-27a/b in the study of a-type ECG and EGCG dimers [40]. As we all know, lipids are important structural components in cell membranes [65]. Notably, with different molecular structures, a-type ECG and EGCG dimers strongly disturbed the structures of cell membranes by decreasing fluidity and hydrophobicity and increasing the permeability of the membrane of 3T3-L1 pre-adipocyte cells, thus displaying significant inhibition on differentiation [40]. EGCG also suppressed 3T3-L1 cell growth via miR-143/MAPK7 pathways [43]. Nonivamide-induced reduction in lipid accumulation was mediated by transient receptor potential cation channel subfamily V member 1 (TRPV1) activation [45]. Although miRNAs are involved in the adipocyte differentiation process, whether they affect membrane structure remains to be intensively studied in natural product therapy.

The activation of C/EBPα and PPARγ is not only necessary for adipocyte differentiation in the early stage but is also crucial for terminal adipocyte differentiation [66]. The evidence suggests that grape seed procyanidin B2 could inhibit pre-adipocyte differentiation and reduce intracellular lipid accumulation by modulating the miR-483/PPARγ axis [42]. Resveratrol reduced the expression of CEBP/α by boosting miR-155, resulting in decreasing lipogenesis [51]. Consistent with these, as shown in Table 2, similar results were also obtained in the research of lycopene by regulating the expression of miR-21 [44]. What is noteworthy is that accompanied with the involvement of multiple miRNAs, *Rosmarinus officinalis* extract significantly reduced triglyceride incorporation during pre-adipocyte maturation in a dose-dependent manner and decreased the expression of cell cycle genes, such as cyclin-dependent kinase 4, cyclin D1, and cyclin-dependent kinase inhibitor 1A [39]. The final and most studied phase of adipocyte differentiation involves terminal differentiation and the induction of a signaling cascade to promote the expression of the genes necessary for adipocyte function [67,68]. The canonical Wnt signaling cascade is an effective approach to suppress adipogenesis [69,70,71]. Recently, investigators found that curcumin repressed 3T3-L1 pre-adipocyte cell adipogenic differentiation by inhibiting the expression of miR-17 and stimulating transcription factor 7-like 2 (TCF7L2), which is the Wnt signaling pathway effector and a direct downstream target of miR-17 [41]. And guarana extract also exerted an anti-adipogenic effect by regulating the Wnt signaling pathway, mediated by miRNAs [32]. In summary, natural products may inhibit adipocyte differentiation and accumulation by regulating miRNAs, which play a crucial role in the process of lipogenesis.

## 4. Effects of Natural Products on Glucose Metabolism Disorders

Glucose metabolism is the basis of metabolism in the body. Metabolic diseases caused by abnormal glucose metabolism, such as diabetes, are the focus and difficulty of current social research due to their progressively expanding populations, complex pathogenesis, drug-maintained recovery, and high expenditure [72,73]. Diabetes is a heterogeneous group of disorders characterized by hyperglycemia due to an absolute or relative deficit in insulin production or action [74]. Under hyperglycemic conditions, reactive oxygen species (ROS) increase, causing cells to activate various abnormal metabolic pathways and inducing oxidative stress [75]. Now that the prevention and treatment of diabetes offers a new avenue, natural products are an increasingly significant area of product development for anti-diabetic drugs. Here, we revealed the effects of natural products on the action of hypoglycemia and the inhibition of oxidative stress by regulating miRNAs (Table 3 and Table 4).

### 4.1. Hypoglycemic Action

**Figure 2:**
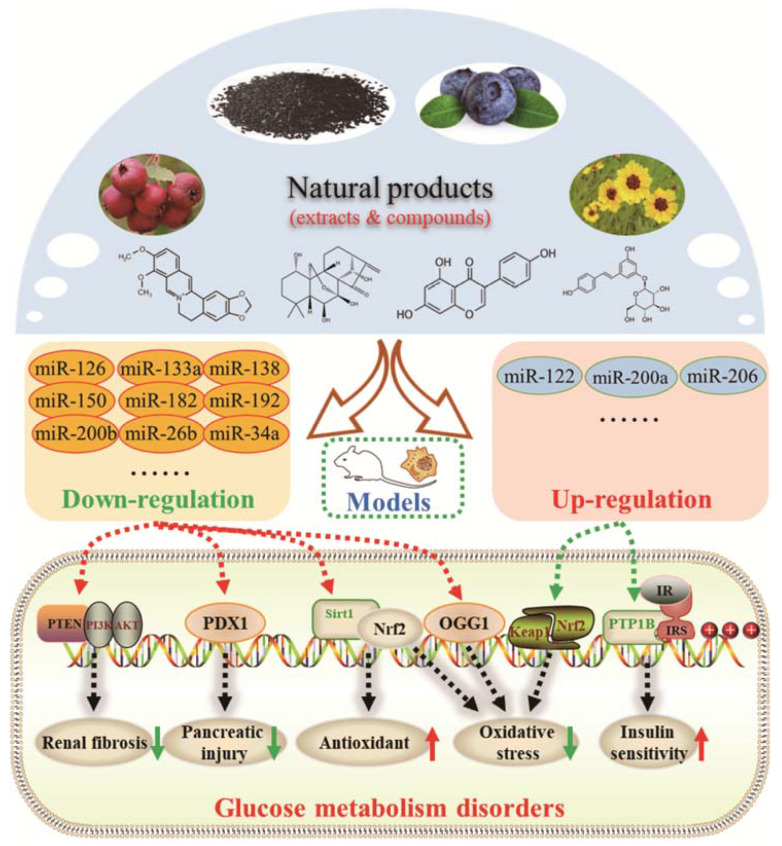
Schematic illustration of the main mechanisms by which natural products improve glucose metabolism disorders mediated by miRNAs. The red arrow means an increase, and the green arrow means a decrease.

At the heart of glucose metabolism is maintaining an equilibrium of glucose concentrations in the blood. So, blood glucose concentration is used as an important indicator of glucose metabolism in the body [91]. Insulin, secreted by β-cells in the pancreas, is the hormone currently known to lower blood glucose in the body. However, it has been now well established from a variety of studies that natural products can reduce blood glucose by acting on insulin (Table 3 and Table 4), while the potential mechanisms of action are diverse (Figure 2).

Protein tyrosine phosphatase 1B (PTP1B) is a major negative regulator of the insulin signaling pathway in metabolism and dephosphorylates insulin receptor (IR) and insulin receptor substrate 1 (IRS1) at tyrosine residues to inhibit the activation of downstream Akt and ERK1/2 signaling cascades [92,93]. Curcumin, however, could induce miR-206 expression, which, in turn, decreased fructose-induced PTP1B overexpression to improve glucose intolerance and insulin sensitivity in fructose-fed rats [83]. Interestingly, the same results were also found in a study on licorice flavonoid, which reversed the decrease of miR-122 induced by the overexpression of PTP1B and abrogated the hepatic insulin resistance induced by an HFD diet [80]. Gypenoside A attenuated the dysfunction of pancreatic β cells by activating pancreatic duodenal homeobox-1 (PDX1) signal transduction via the inhibition of miR-150 in HFD-fed mice [86]. Vaccarin, an active flavonoid glycoside extracted from vaccariae semen, reduced blood glucose, increased glucose and insulin tolerance, and relieved glucose metabolism disturbances in STZ/HFD-induced type 2 diabetes mellitus (T2DM) mice by regulating miR-34a expression [90]. *Coreopsis tinctoria nutt* extract treatment also showed the effect of lowering fasting blood by inhibiting the expression of miR-192 and miR-200b [78]. From the above, insulin plays an important role in blood glucose stability, and importantly, both low-insulin secretion and insulin resistance can lead to blood glucose disorders. This can, however, be reversed, at least in part, by natural products through miRNA cascades.

In addition, by using hierarchical clustering analysis, the miRNA expression patterns, as well as miRNA microarray analysis, are shown in Table 3. It was macroscopically discovered that various miRNAs participated in the hypoglycemic process in *Alpinia oxyphylla* extract treatment [76]; however, the underlying mechanism of action is not yet clear.

### 4.2. Restraining Effects on Oxidative Stress

Insulin signaling has been one of the most important and highly studied metabolic hormones for glucose metabolism homeostasis. ROS are usually produced in the process of biological oxidation and energy conversion in mitochondria; however, the enhancement of ROS induced by a hyperglycemic environment disrupts the balance between ROS and the antioxidant system, resulting in oxidative stress, which subsequently induces insulin resistance and pancreatic β cell dysfunction via their ability to activate stress-sensitive signaling pathways [94]. Therefore, oxidative stress has been defined as a disturbance in the dynamic balance between ROS generation and antioxidant capacity [95], and ROS generation is also regarded as a marker of oxidative stress, which can lead to pancreatic β cell dysfunction and peripheral insulin resistance, hence, resulting in glucose metabolic disorders [95,96]. A growing number of studies have shown that natural products can inhibit oxidative stress by monitoring various miRNAs (Table 3 and Table 4).

Insulin resistance in the brain is a specific form of T2DM; however, *Nigella sativa* oil has a possible benefit as a disease-modifying agent for insulin resistance in the brain by suppressing oxidative stress and enhancing the brain insulin signaling pathway; multiple miRNAs are involved in this process, especially miR-34a and miR-26b [81]. This also supports the view that insulin resistance partly originates from oxidative stress [97]. In addition, in experiments on blueberry anthocyanin extract, *Crataegus persica* extract, polydatin, and sodium tanshinone IIA sulfonate treatments all decreased the ROS level and alleviated the oxidative stress induced by different high-concentration glucose environments [77,79,88,89]. Pieces of evidence have been accumulating regarding Sirt1 playing an important role in the cellular redox balance and resistance to oxidative stress [98,99]. Furthermore, Sirt1 can regulate nuclear factor erythroid 2-related factor 2 (Nrf2) to regulate the transcription of pro- and anti-oxidant enzymes, subsequently affecting the cellular redox state [100]. Dioscin, a natural steroid saponin isolated from various herbs [101], significantly decreased the formation of ROS and suppressed oxidative stress by regulating the miR-34a/Sirt1/Nrf2-mediated pathway in vivo and in vitro [84]. These results were consistent with the data obtained from a study on genistein which could raise anti-oxidative ability through the upregulation of Sirt1 via inhibiting miR-34a in in vitro experiments [85]. Additionally, astragaloside IV also increased cellular antioxidant capacity and alleviated high-glucose-induced cell damage, the potential mechanism of which probably owes credit to the enhanced Sirt1/Nrf2 activity induced by miR-138 [82]. Oridonin, a diterpenoid isolated from *Rabdosia rubescens*, attenuated hydrogen peroxide-induced oxidative stress by altering miRNAs expression; statistically, six miRNAs were upregulated, and 15 miRNAs were downregulated by using microarray analysis [87].

In general, as a negative effect produced by excess ROS in the body, oxidative stress is an important common pathogenesis of pancreatic β cell injury, which, in turn, affects insulin secretion. As antioxidants, natural products are effective at removing excess free radicals from the body and regulating oxidative stress (Figure 2). From the perspective of drug development, the study of oxidative stress can help to shed further light on the pathogenesis of abnormal glucose metabolism and provide a theoretical basis for the prevention and treatment of glucose metabolism disorders and their complications.

## 5. Effects of Natural Products on Cardiovascular Diseases

Cardiovascular disease is one of the major causes of death worldwide, with morbidity and mortality rising year by year [102]. Cardiovascular disease, also known as circulatory disease, is a series of diseases that involve the circulatory systems [103]. Moreover, abnormal lipid metabolism and glucose metabolism are important factors in the process of cardiovascular disease [104]. The changes experienced by using natural products in recent years are still unprecedented. Existing natural product studies have shown therapeutic effects on myocardial cell injury and protective effects on vascular endothelial cells via miRNA-mediated signaling pathways (Table 5 and Table 6).

### 5.1. Therapeutic Effects on Myocardial Cell Injury

Cardiomyocyte injury is closely related to the development of cardiovascular diseases, such as myocardial failure, myocardial ischemia, cardiac fibrosis, and myocardial infarction [105,106,107,108]. Increasing evidence suggests that natural products protect cardiomyocytes from various injuries by managing the expression of miRNAs.

Tanshinone IIA, the active ingredient isolated from the rhizome of the Chinese herb *Salvia miltiorrhiza* (also known as “*Danshen*” in Chinese), is an effective cardioprotective agent. Latterly, it was indicated that tanshinone IIA could protect cardiomyocytes from ischemic and hypoxic damage, which was based on downregulating the expression of miR-1 and upregulating the expression of miR-133 by activating the P38 MAPK and ERK1/2 signal pathway, respectively [109,110]. It could also modulate the overexpressed miR-1 by regulating serum response factor (SRF) [111], a transcriptional regulator of muscle-specific and growth-regulated genes, which may lower the risk of sudden cardiac death [112]. As we mentioned in the context of glucose metabolism disorders, ROS accumulation is not only an indicator of oxidative stress injury but also a marker of cardiomyocyte damage. The interventional treatment of gypenoside A, resveratrol, and portulaca oleracea extract significantly reduced ROS production and attenuated myocardial injuries; meanwhile, it exerted cardio-protective effects via miRNA-mediated signaling pathways [113,114,115], whereas dioscin inhibited myocardial oxidative insult and alleviated doxorubicin-induced cardiotoxicity via the miR-140/Sirt2/Nrf2 signaling pathway [116].

There is evidence that apoptosis is involved in the development of myocardial infarction and heart failure [117]. A test study indicated that resina draconis treatment inhibited the endoplasmic reticulum-induced apoptosis of myocardial cells via regulating the miR-423/ERK signaling pathway in a tree shrew myocardial IR model [118], whereas salvianolate treatment blocked apoptosis during myocardial infarction by downregulating miR-122 [119]. As a heart-healthy compound, it was uncovered for the first time that resveratrol (100 mg/kg/day) treatment could suppress the apoptosis of myocardium in cold-treated mice by inhibiting miR-328 expression [120]. Furthermore, curcumin could also protect cardiomyocytes against hypoxia-induced apoptosis by modulating specific protein 1, which participated in co-ordinating the transactivation of survivin, a crucial gene in regulating cell apoptosis [121], which is regulated by miR-7a/b [122]. Some other natural products or their extracts, such as puerarin, ginsenoside Rb1, theaflavin, astragalus root dry extract, and *Crataegus persica* extract, also contributed to the protection of various types of myocardial injury and exhibited cardio-protective effects by controlling miRNA cascades, respectively [79,123,124,125,126].

**Figure 3:**
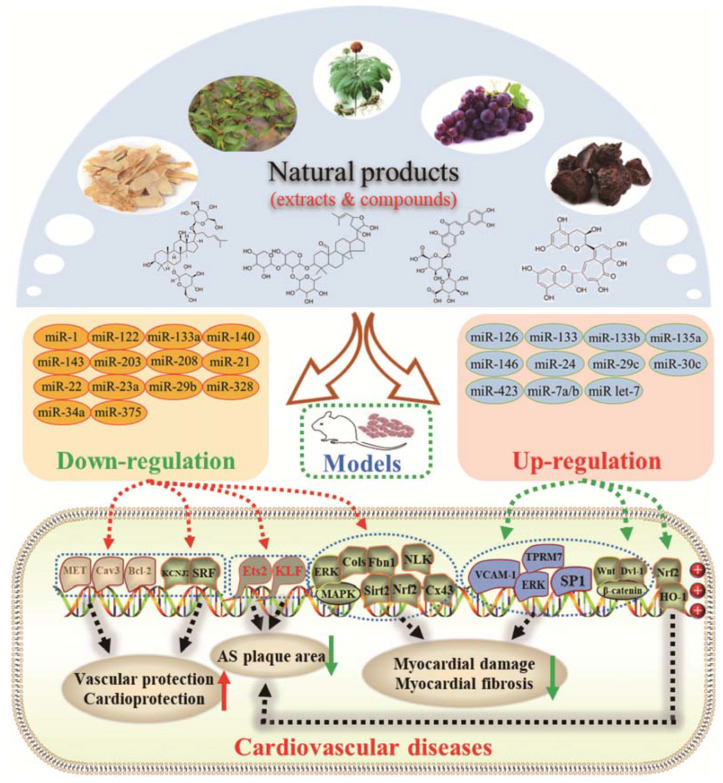
Schematic illustration of the main mechanisms by which natural products improve cardiovascular diseases mediated by miRNAs. The red arrow means an increase, and the green arrow means a decrease.

Myocardial fibrosis, a common cardiac response in a variety of forms of damage, is characterized by excessive collagen deposition and extra-cellular matrix accumulation [127]. However, celastrol, a quinone methide triterpene isolated from the root extracts of *Tripterygium wilfordii* (*Thunder god vine*) [128], could reverse these undesirable phenomena induced by downregulating miR-21 expression and inhibiting MAPK/ERK signaling in transverse aortic constriction mice [129]. Similarly, Luteolin-7-diglucuronide, a naturally occurring flavonoid glycoside found in the leaves of *Basil* or *Verbena officinalis*, also attenuated isoproterenol-induced myocardial fibrosis both at the histo-pathological and molecular levels, accompanied by regulating the expression of miRNAs, including miR-29c, miR-39c, miR-133b, and miR-21 via the TGF-β signaling pathway [130]. Astragaloside IV inhibited cardiac fibrosis by targeting the miR-135a-TRPM7-TGF-β/Smads signaling pathway [131]. Identical results were also detected in the study of panax notoginseng saponins [18]. Myocardial damage and the consequent fibrotic alterations impair the normal heart architecture and cause cardiac dysfunction (Figure 3). Fortunately, these studies provide new insight into the molecular mechanisms of natural products in the studies of cardiovascular diseases.

**Table 5:** The effects of natural products (extracts) on cardiovascular diseases.

Natural Products (Extracts)	Relevant miRNAs	Dose	Administration Methods	Experimental Models	Targets	Observed Effects	References
Açaí and red muscadine grape polyphenolics	miR-126↑	5–20 mg GAE/L for 30 min	Cell culture	HUVECs	VCAM-1	● Protected HUVEC against glucose-induced oxidative stress and inflammation; ● Inhibited gene expression of adhesion molecules and NF-κB activation.	[132]
Astragalus root dry extract	miR-1↓	20 mg/kg/d for 7 days	Intraperitoneal injection	CVB3-treated mice	Cx43	● Prevented the increase of immune cell infiltration and arrhythmia.	[90]
*Crataegus persica* extract	miR-126↑	300 mg/kg/d for 10 weeks	Gavage	Diabetic rats	/	● Decreased elevated levels of renal oxidative stress, glomerular filtration rate, insulin sensitivity, and pathological score; ● Ameliorated myocardial ischemia-reperfusion-induced renal injury.	[79]
Panax notoginseng saponins	miR-29c↑	150 mg/kg/d for 20 days	Intraperitoneal injection	ISO-treated mice	Cols, Fbn1	● Alleviated ISO-induced myocardial injury and fibrotic alterations; ● Cardioprotective effects.	[18]
Portulaca oleracea extract	miR-146↑ miR-let-7↑	300 mg/kg/d for 35 days	Gavage	Lipopolysaccharide treated mice	/	● Protected from LPS-induced neuroinflammation and memory decline through antioxidant and anti-inflammatory effects.	[115]
Resina draconis	miR-423↑	0.25, 0.5 and 1.0 mg/mL	Intramuscular injection	Ischemia-reperfusion tree shrew	ERK	● Reduced the infarct size, enhanced the superoxide dismutase expression, and downregulated the malondialdehyde concentration; ● Suppressed the ischemia-reperfusion-induced apoptosis.	[118]
Salvianolate	miR-122↓	12, 24 and 48 mg/kg/d for 2 weeks	Intraperitoneal injection	Myocardial infarction rats	/	● Induced the anti-apoptosis mechanism of cardiomyocytes.	[119]
Xiaoxianggou	miR-203↓	10, 20, and 40 g/kg, two times one week for 16 weeks	Gavage	Endogenous high Ang II ApoE ^−/−^ mice	Ets2	● Reduced the atherosclerotic plaque area and serum autoantibodies against oxLDL.	[133]

The up arrow means an increase, and the down arrow means a decrease.

**Table 6:** The effects of natural products (compounds) on cardiovascular diseases.

Natural Products (Compounds)	Relevant miRNAs	Dose	Administration Methods	Experimental Models	Targets	Observed Effects	References
Astragaloside IV 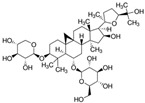	miR-135a↑	10 mg/kg/d for 9 days	Gavage	Isoproterenol-treated rats	TPRM7	● Inhibited cardiac fibrosis by targeting the miR-135a-TRPM7-TGF-β/Smads pathway.	[131]
Berberine 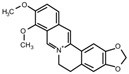	miR-133a↓	1.0 g/kg/d for 8 weeks	Gavage	STZ-induced diabetic rats	/	● Improved vascular dementia; ● Improved impairments of learning and memory.	[134]
Celastrol 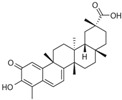	miR-21↓	1 mg/kg/d for 3 and 12 weeks	Intraperitoneal injection	Transverse aortic constriction mice	ERK1/2	● Attenuated TAC-induced cardiac hypertrophy; ● Reduced the increased collagen deposition and downregulated α-smooth muscle actin; ● Attenuated pathological myocardial fibrosis.	[129]
Curcumin 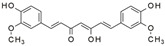	miR-7a/b↑	50 mg/kg/d for 1 week	Gavage	Myocardial infarction mice	SP1	● Reduced the infarct size; ● Protected against hypoxia-induced cardiac myocytes apoptosis.	[122]
Dioscin 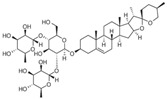	miR-140↓	Rats treated with 60, 30, and 15 mg/kg/d for 7 days; mice treated with 80, 40, and 20 mg/kg/d for 7 days	Gavage	Doxorubicin-treated rats and mice	Sirt2, Nrf2	● Improved histopathological and electrocardiogram changes; ● Inhibited myocardial oxidative insult; ● Alleviated doxorubicin-induced cardiotoxicity.	[116]
Emodin 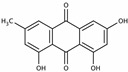	miR-126↑	40 mg/kg/d for 7 weeks	Gavage	Balloon-injured carotid artery rats	Wnt4, Dvl-1, β-catenin	● Prevented intimal thickening via Wnt4/Dvl-1/β-catenin signaling pathway.	[135]
Ginsenoside Rb1 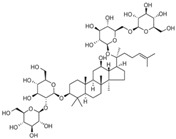	miR-208↓	40 μM for 24 h	Cell culture	Neonatal rat cardiomyocytes	NLK	● Ameliorated cardiomyocytes apoptosis; ● Protected cardiomyocytes injuries.	[124]
Ginsenoside Rg1 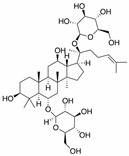	miR-23a↓	150 nM for 24 h	Cell culture	HUVECs	MET	● Increased MET protein expression in a time-dependent manner; ● Induced angiogenesis by the inverse regulation of MET tyrosine kinase receptor expression.	[136]
Gypenoside A 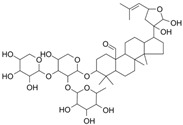	miR-143↓	100 mg/kg 1 h before I/R administration	Gavage	Myocardial I/R injured rats	/	● Cardio-protective effect; ● Attenuated I/R-induced injures; ● Activated AMPK signaling.	[114]
Luteolin-7-diglucuronide 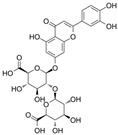	miR-29c, miR-30c, miR-133b: ↑; miR-21↓	40 mg/kg/d for 11 days	Intraperitoneal injection	Isoproterenol-treated mice	Cols, elastin and Fbn1; CTGF	● Attenuated ISO-induced myocardial fibrosis; ● Suppresses ISO-induced oxidative stress and upregulation of NADPH oxidase; ● Reduced myocardial fibrotic lesions.	[130]
Puerarin 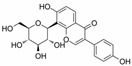	miR-22↓	100 μM for 20 days	Cell culture	The mES cell line D3 and its transgenic cell line αPIG (clone 44)	Cav3	● Improved the myofibrillar alignment and sarcomere development; ● Promoted the development of t-tubules; ● Upregulated the t-tubules biogenesis-related genes.	[123]
Resveratrol 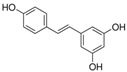	miR-34a↓	20, 50 or 100 mΜ for 48 h	Cell culture	Rat heart-derived H9c2 cells	/	● Enhanced cell viability; ● Reduced cell apoptosis; ● Protective effect on cardiomyocytes.	[113]
	miR-328↓	100 mg/kg/d for 8 weeks	Gavage	Cold-treated mice	/	● Inhibited alteration of cardiac structure; ● Improved ultrastructure of myocardium; ● Improved cardiac function; ● Suppressed cold-induced hypertension; ● Suppressed apoptosis of myocardium.	[120]
	miR-29b↓	0.1 mg/mL for 2 months	Drinking	Fbn1^C1039G/+^ Marfan mice	Bcl-2	● Promoted elastin integrity and smooth muscle cell survival; ● Inhibited aortic root dilatation.	[137]
Sodium Tanshinone IIA Sulfonate 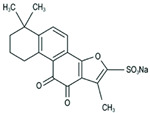	miR-133a↓	10 mg/kg/d for 3 weeks	Gavage	PAD mice	/	● Improved perfusion recovery, increased capillary densities, decreased ROS level in the ischemic hindlimb in diabetic mice; ● Improved angiogenesis via inhibiting miR-133a expression and increasing GCH-1 protein levels.	[89]
Tanshinone IIA 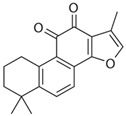	miR-375↓	10 mg/kg for 20 weeks	Intraperitoneal injection	HFD-fed ApoE-/- mice	KLF4	● Attenuated atherosclerosis.	[138]
	miR-1↓	20 mg/kg/d for 3 months	Gavage	Myocardial infarction rats	Cx43	● Improved the hemodynamic parameters; ● Regulated P38 MAPK pathway; ● Relieved ischemia-induced injury.	[109].
	miR-1↓	10 mg/kg/d for 3 months	Gavage	Myocardial infarction rats	KCNJ2, SRF	● Raised survival rates; ● Ameliorated dysfunction of IK_1_; ● Suppressed ischemic arrhythmias and cardiac mortality.	[111]
	miR-133↑	10 µM for 30 min	Cell culture	Neonatal rat cardiomyocytes	ERK1/2	● Increased cell viability; ● Protected cell against apoptosis; ● Activated MAPK ERK1/2 signaling.	[110]
Theaflavin 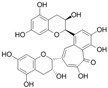	miR-24↑	5, 10 mg/kg/d for 12 weeks	Gavage	HFD-fed ApoE^−/−^ mice	Nrf2, HO-1	● Promoted the activities of antioxidant enzymes (SOD, CAT, and GSH-Px); ● Inhibited the formation of atherosclerotic plaque and the process of histological alterations in the aorta.	[125]

The up arrow means an increase, and the down arrow means a decrease.

### 5.2. Protective Effects on Vascular Endothelial Cells

Vascular endothelial cells form the interface between blood and tissues and are involved in physiological and pathological processes, including cardiovascular diseases [139,140]. Massive cardiovascular diseases lead to various degrees of vascular endothelial injury, which, in turn, exacerbates cardiovascular diseases. Vascular endothelial dysfunction is closely related to the development of cardiovascular diseases [141]. Vascular endothelial cells are not only the target organs of cardiovascular diseases but also the new target organs of many drugs [142,143]. Therefore, improving vascular endothelial function has been an important aspect of anti-cardiovascular drug development in recent years. Obviously, as shown in Table 5 and Table 6, natural products that regulate the expressions of miRNAs may be a better choice.

Polyphenolics from açaí and red muscadine grape ameliorated human umbilical vascular endothelial cell (HUVEC) injury by inhibiting the gene expression of adhesion molecules, including vascular cell adhesionmolecule-1 (VCAM-1), which is targeted by miR-126 [132]. Ginsenoside-Rg1, which is derived from *Ginseng*, was considered an agent that promotes angiogenesis because the decreased expression of miR-23a negatively regulates the angiogenic activities of HUVEC in vitro [136]. Sodium tanshinone IIA sulfonate treatment improved angiogenesis by regulating the miR-133a/GCH-1 signaling pathway in experimental peripheral arterial disease (PAD) in diabetes [89].

In addition, vascular endothelial cell injury is the initial stage of atherosclerosis [144]. According to the research, xiaoxianggou, the dried root and stem of *Ficus pandurata hance var. angustifolia Cheng*, *Ficus panduram hane var. hoiophylla Migo,* and *Ficus erecta thunb. var. bcecheyana King*, could reduce the area of atherosclerotic plaque by elevating miR-203 expression and reducing the expression of E26 oncogene homolog 2 (Ets2) [133], which could promote further lesion destabilization by directly affecting endothelial cell function, promoting vessel leakage and expansive neovascular growth from the adventitia into the intimal area [145]. Intimal hyperplasia has long been a major problem plaguing vascular surgery. The proliferation of vascular smooth muscle cells (VSMCs) is an important factor that causes intimal thickening [146]. Nevertheless, resveratrol improved atherosclerosis by reducing higher collagen deposition and promoting elastin integrity and VSMC survival mediated by reducing the expression of miR-29b in the Fbn1^C1039G/+^ Marfan mouse model [137]. There were similar results in the study on emodin; interestingly, miR-126 participated in this process by mediating the Wnt4/Dvl-1/β-catenin signaling pathway in balloon-injured carotid artery rats [135]. Berberine improved vascular dementia in diabetes, which is possibly related to the suppression of miR-133a ectopic expression in endothelial cells [134]. A key regulatory role for Krüppel-like factor 4 (KLF4) in vascular function has been shown in vitro and in vivo, and KLF4 deficiency is associated with atherothrombosis [147,148,149,150]. Tanshinone IIA harmonized the crosstalk of autophagy and polarization in macrophages via activating KLF4 mediated by miR-375 to attenuate atherosclerosis [138]. These studies set out our vision of the protective effect of natural products on vascular endothelial cells by the regulation of multiple miRNAs (Figure 3), and provide molecular evidence for further studies on natural products as novel anti-cardiovascular therapies.

## 6. Conclusions

In all the studies reviewed here, while natural products have provided new insights into the treatment of metabolic diseases by regulating miRNA cascades and have revealed anti-obesity, anti-diabetes, and anti-cardiovascular disease functions, as well as demonstrating a rich source of therapeutic agents, there are still some pressing issues that need to be addressed. Primarily, the mechanisms of metabolic diseases and the correlations between them are complex and still require systematic and in-depth studies beyond all doubt. Moreover, the plentiful miRNAs existing in our bodies often act together with their cluster members or other miRNAs [151]; hence, the complicated regulatory network of miRNAs can also not be ignored in natural product treatments. In addition, with a view of providing better clues for drug development, there are many natural products that have not yet been discovered in nature, of which the active substances and their effects on miRNAs still need to be further investigated.

## Figures and Tables

**Table 1:** The effects of natural products (extracts) on lipid metabolism disorders.

Natural Products (Extracts)	Relevant miRNAs	Dose	Administration Methods	Experimental Models	Targets	Observed Effects	References
*Averrhoa carambola* free phenolic extract	miR-33↓ miR-34a↓	10, 20, 30 g/kg/d for 8 weeks	Gavage	db/db mice	/	● Reduced liver TG; ● Inhibited the signal transduction of hepatic lipogenesis; ● Exhibited a potent hepatic steatosis-relieving effect.	[20]
Cerasus humilis polyphenol extract	miR-7a/b↓	40 μg/mL for 48 h; 250 g/kg/day for 12 weeks	Cell culture; gavage	3T3-L1 pre-adipocyte cells; obese mice	Sirt1, Prdm16	● Reduced body weight; ● Improved abnormal serum lipid and glucose levels; ● Inhibited adipocyte differentiation; ● Reduced fat accumulation by mitigating fat deposition, inflammation, and oxidation.	[21]
Citrus peel flavonoids	miR-33↓ miR-122↓	10 μg/mL for 0.5, 1, 3 and 6 h	Cell culture	Oleic acid-treated HepG2 cells	FAS, CPT1a	● Attenuated intracellular lipid accumulation.	[22]
Coffee polyphenols	miR-122↑	2.5 × 10^−4^%; diet containing 0.5% or 1.0% coffee polyphenols for 15 weeks	Cell culture; diet	Hepa 1-6 cells; HFD-fed mice	SREBP1c	● Activated AMPK; ● Enhanced energy metabolism; ● Reduced lipogenesis; ● Reduced body weight gain, abdominal and liver fat accumulation.	[23]
Ginger extract	miR-21↓ miR-132↓	Diet containing 0.8% ginger extract for 10 weeks	Diet	HFD-fed rats	/	● Lowered body weight and white adipose tissue mass; ● Reduced serum and hepatic lipid levels; ● Enhanced AMPK activity; ● Ameliorated obesity and inflammation.	[24]
Grape seed proanthocyanidins extract	miR-33a↓ miR-122↓	5, 25, 50 mg/kg for 3 weeks	Gavage	HFD–induced obese rats	ABCA1; FAS, PPARβ/δ	● Hypolipidemic; ● Decreased total liver fat.	[16]
	miR-33a↓ miR-122↓	5, 15, 25, 50 mg/kg for 3 weeks	Gavage	Healthy Wistar rats	ABCA1; FAS	● Improved postprandial hyperlipemia; ● Increased liver cholesterol efflux to HDL formation; ● Reduced fatty acid synthesis.	[25]
	miR-33↓ miR-122↓	10, 25, 50, or 100 mg/L for 0.5, 1, 3, or 5 h; 250 mg/kg for 1 or 3 h	Cell culture; gavage	FAO cells; Wistar rats	ABCA1; FAS	● Hypolipidemic; ● Reduced lipogenesis; ● Increased liver cholesterol efflux to HDL formation.	[26]
	miR-33a↓ miR-122↓	25 mg/kg for 3 weeks	Gavage	Dyslipidemic obese rats	ABCA1, CPT1a; FAS, PPARβ/δ	● Improved dyslipidemia; ● Decreased total liver fat.	[27]
	miR-96↓	200 mg/kg/day for 180 days	Diet	HFD-fed mice	mTOR, FOXO1	● Decreased the weight gain, serum levels of triglycerides, total cholesterol, and low-density lipoprotein cholesterol but increased high-density lipoprotein cholesterol; ● Clearance of lipid accumulation.	[28]
	miR-33↓ miR-122↓	250 mg/kg once	Gavage	HFD-fed grass carp	/	● Decreased TG accumulation by reducing de novo lipogenesis and enhancing lipolysis and β-oxidation.	[29]
Green tea extract	miR-34a↓ miR-194↑	500 mg/kg for 12 weeks (5 days/week)	Gavage	HFD-fed mice	Sirt1, PPARα, INSIG2; HMGCS, APOA5	● Protected against NAFLD development by altering lipid metabolism, increasing gene expression involved in triglycerides and fatty acid catabolism, and decreasing uptake and lipid accumulation.	[30]
	miR-335↓	500 mg/kg for 12 weeks (5 days/week)	Gavage	HFD-fed mice	FOXO1, GSK3β	● Reduced weight gain, adiposity and inflammation; ● Increased energy expenditure; ● Improved insulin sensitivity.	[31]
Guarana extract	miR-27b↓ miR-34b↓ miR-760↓	150 µg/mL for 48 h	Cell culture	3T3-L1 pre-adipocyte cells	Wnt3a, Wnt1, Wnt10b	● Anti-adipogenic effect.	[32]
Lychee pulp phenolics	miR-33↓ miR-122↓	500 mg/kg for 10 weeks	Gavage	HFD-fed mice	ABCA1, ABCG1, NPC1; FAS, ACC1, ACC2, SCD1, ACLY	● Hypolipidemic; ● Repressed fatty acid synthesis and promoting fatty acid β-oxidation and cholesterol efflux in the liver; ● Decreased body fat accumulation; ● Ameliorated lipid metabolism.	[33]
Mulberry fruit extract	miR-33↓	Diet containing 0.4% mulberry fruit extract for 4 weeks	Diet	High cholesterol/cholic acid diet-fed rats	/	● Promoted serum high-density lipoprotein cholesterol levels; ● Decreased serum and hepatic cholesterol, serum low-density lipoprotein cholesterol, and fecal bile acid levels.	[34]
Mulberry leaf extract	miR-34a↓	3 mg/mL for 24 h	Cell culture	Glucolipotoxicity-induced HepG2 cells	Sirt1	● Reduced liver fat accumulation; ● Decreased inflammatory responses and steatohepatitis; ● Exerted anti-glucolipotoxicity effects.	[35]
Moringa oleifera leaf extract	miR-21a↓ miR-103↓ miR-122↓ miR-34a↓	9.375 mg/d for 8 weeks	Gavage	HFD-fed mice	/	● Improved ITT and decreased SREBP1c hepatic protein, while Sirt1 increased; ● Reduced insulin resistance, de novo lipogenesis, hepatic inflammation, and ER stress; ● Prevented progression of liver damage in a model of NASH.	[36]
Portulaca oleracea extract	miR-122↓	25, 50, 100 mg/kg/d for 7 days	Gavage	Acute alcoholic liver injury rats	/	● Reduced the ethanol-elevated serum level of ALT, AST, ALP, and TG; ● Enhanced activities of SOD and GSH-Px; ● Decreased content of NO and MDA; ● Increased antioxidant capacity; ● Relieved the inflammatory injury; ● Improved the lipid metabolism disorder.	[37]
	miR-33↓ miR-34a↓	Diet containing 0.8% portulaca oleracea L. extract for 4 weeks	Diet	High-cholesterol diet-fed rats	/	● Improved serum, liver, and fecal lipid profiles; ● Promoted cholesterol efflux and bile acid synthesis; ● Enhanced hepatic AMPK activity.	[38]
*Rosmarinus officinalis* extract	miR let-7f-1↑	30 μg/mL for 35 days	Cell culture	Human primary omental pre-adipocytes and adipocytes	/	● Decreased triglyceride accumulation; ● Increased glycerol release; ● Stimulated lipolytic activity in differentiating pre-adipocytes and mature adipocytes; ● Modulated the adipocyte life cycle at different levels.	[39]

The up arrow means an increase, and the down arrow means a decrease.

**Table 2:** The effects of natural products (compounds) on lipid metabolism disorders.

Natural Products (Compounds)	Relevant miRNAs	Dose	Administration Methods	Experimental Models	Targets	Observed Effects	References
A-type ECG and EGCG dimers 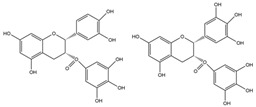	miR-7a/b↑	ECG dimer: 20 μg/mL for 1–8 days; ECGG dimer: 60 μg/mL for 1–8 days	Cell culture	3T3-L1 pre-adipocyte cells	PPARγ	● Inhibited pre-adipocyte differentiation; ● Reduced intracellular lipid accumulation; ● Blocked MCE process; ● Decreased the fluidity and hydrophobicity and increased the permeability of membrane.	[40]
Curcumin 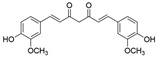	miR-17↓	2 μM or 10 μM for 6 h;	Cell culture	3T3-L1 pre-adipocyte cells; HFD-fed mice	TCF7L2	● Inhibited adipocyte differentiation and adipogenesis; ● Stimulated the Wnt signaling pathway.	[41]
Grape seed procyanidin B2 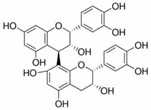	miR-483↓	150 μg/mL for 48 h	Cell culture	3T3-L1 pre-adipocyte cells	PPARγ	● Inhibited pre-adipocyte differentiation; ● Reduced intracellular lipid accumulation.	[42]
EGCG 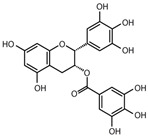	miR-143↑	50 μM for 24 h	Cell culture	3T3-L1 pre-adipocyte cells	MAPK7	● Inhibited 3T3-L1 cell growth.	[43]
Lycopene 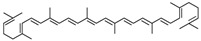	miR-21↑	50 μM for 24 h; diet containing 0.05% lycopene for 8 weeks	Cell culture; gavage	Hepa 1–6 cells; HFD-fed mice	FABP7	● Lowered body weight; ● Inhibited intracellular lipid accumulation; ● Protected against HFD-induced hepatic steatosis.	[44]
Nonivamide 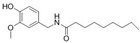	miR let-7d↑	1 μM for 12 days	Cell culture	3T3-L1 pre-adipocyte cells	PPARγ	● Impaired adipogenesis; ● Reduced mean lipid accumulation; ● Activated TRPV1.	[45]
Oleanolic acid 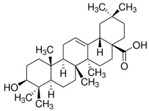	miR-98↑	10 mM for 6, 12, 24 h; 20 mg/kg for 4 weeks	Cell culture	HFD-fed mice; db/db mice	PGC1β	● Hypolipidemic.	[46]
Persimmon tannin 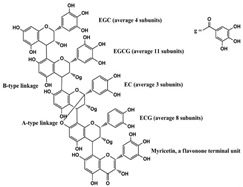	miR-27↑	20, 40, or 60 μg/mL for 1–8 days	Cell culture	3T3-L1 pre-adipocyte cells	PPARγ, C/EBPα	● Inhibited pre-adipocyte differentiation; ● Reduced intracellular lipid accumulation; ● Delayed MCE process.	[47]
Pseudoprotodioscin 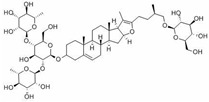	miR-33a/b↓	25 μM for 24 h	Cell culture	Human HepG2 cells and THP-1 monocytic cells	SREBP1c, SREBP2	● Promoted the cholesterol effluxion.	[48]
Resveratrol 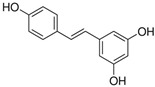	miR-103↓ miR-107↓ miR-122↓	30 mg/kg for 6 weeks	Diet	Obesogenic diet-fed rats	SREBP1; SREBP1, CPT1a; FAS	● Reduced obesogenic diet-induced hepatic steatosis; ● Activated AMPK.	[49]
	miR-539↑	30 mg/kg for 6 weeks	Diet	Obesogenic diet-fed rats	SP1	● Inhibited de novo lipogenesis.	[50]
	miR-155↑	25 μM for 1–8 days	Cell culture	3T3-L1 pre-adipocyte cells	CEBP/α	● Inhibited adipogenesis.	[51]
Zerumbone 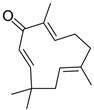	miR-46b↓	25 μM for 48 h; diet containing 0.025% zerumbone for 8 weeks	Cell culture; diet	3T3-L1 fibroblasts; HFD-fed mice	Sirt1	● Induced AMPK activation and phosphorylation of acetyl-CoA carboxylase; ● Ameliorated diet-induced obesity and inhibited adipogenesis.	[52]

The up arrow means an increase, and the down arrow means a decrease.

**Table 3:** The effects of natural products (extracts) on glucose metabolism disorders.

Natural Products (Extracts)	Relevant miRNAs	Dose	Administration Methods	Experimental Models	Targets	Observed Effects	References
*Alpinia oxyphylla* extract	miR-let-7k, miR-378d: ↑; miR-129, miR-21a, miR-29c, miR-203, miR-7a: ↓	50 mg/kg/d for 8 weeks	Gavage	DB/DB and db-/db- mice	/	● Lowered concentrations of blood glucose; ● Changed the expressions of specific miRNAs.	[76]
Blueberry anthocyanins extract	miR-182↓	200 mg/kg/d for 6 days	Gavage	STZ-induced diabetic rats	OGG1	● Restored the increase of apoptosis, ROS level, and ERS induced by high-concentration glucose.	[77]
*Coreopsis tinctoria nutt* extract	miR-192↓ miR-200b↓	300 mg/kg/d for 10 weeks	Gavage	db/db mice	ZEB2, PTEN	● Decreased body weight, fasting blood glucose, and 24 h urinary albumin excretion; alleviated kidney damage; ● Modulated the activity of the PTEN/PI3K/AKT pathway to reduce the degree of renal fibrosis.	[78]
*Crataegus persica* extract	miR-126↓	300 mg/kg/d for 10 weeks	Gavage	Diabetic rats	Nrf2	● Decreased elevated levels of renal oxidative stress, glomerular filtration rate, insulin sensitivity, and pathological score.	[79]
Licorice flavonoid	miR-122↑	30 mg/kg for 5 weeks, 5 times per week	Gavage	HFD-fed mice	PTP1B	● Reduced blood glucose; ● Restored IR and IRS1/2 tyrosine phosphorylation and insulin signaling; ● Abrogated hepatic insulin resistance induced by HFD diet.	[80]
*Nigella sativa* oil	miR-34a↓ miR-26b↓	2.0 mL for 21 days	Gavage	Diabetic rats	/	● Suppressed oxidative stress; ● Improved insulin resistance and insulin signaling pathway.	[81]

The up arrow means an increase, and the down arrow means a decrease.

**Table 4:** The effects of natural products (compounds) on glucose metabolism disorders.

Natural Products (Compounds)	Relevant miRNAs	Dose	Administration Methods	Experimental Models	Targets	Observed Effects	References
Astragaloside IV 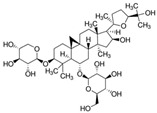	miR-138↓	25 mM for 48 h	Cell culture	High glucose cultured retinal pigment epithelial cells	Sirt1, Nrf2	● Alleviated high glucose-induced RPE cell damage; ● Increased Sirt1/Nrf2 activity and cellular antioxidant capacity; ● Alleviated ferroptosis; ● Decreased cell death.	[82]
Curcumin 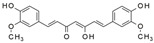	miR-206↑	15, 30, and 60 mg/kg/d for 6 weeks	Gavage	Fructose-fed rats	PTP1B	● Improved insulin signaling; ● Improved glucose intolerance and insulin sensitivity.	[83]
Dioscin 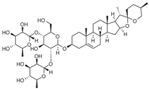	miR-34a↓	50, 100, and 200 ng/mL for 12 h; 10, 20, and 40 mg/kg for 12 days; 15, 30, and 60 mg/kg/d for 10 days	Cell culture; gavage	NRK-52E and HK-2 cells; Wistar rats; C57BL/6J mice	Sirt1	● Decreased the ROS levels; ● Suppressed oxidative stress.	[84]
Genistein 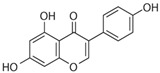	miR-34a↓	1000 nM for 6 h	Cell culture	HUVECs	Sirt1	● Restrained ROS and MDA production; ● Ameliorated the inhibitory effect on SOD, CAT, GSH, GPx activity; ● Suppressed oxidative stress.	[85]
Gypenoside A 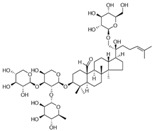	miR-150↓	50 or 100 mg/kg/d for 12 weeks	Gavage	HFD-fed mice	PDX1	● Alleviated pancreatic impairments; ● Improved the dysfunction of β pancreatic cells.	[86]
Oridonin 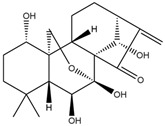	miR-1305, miR-152, miR-182, miR-29b, miR-4298, miR-939: ↑; miR-1246, miR-1268, miR-1290, miR-135a, miR-181b, miR-1973, miR-210, miR-30c, miR-30e, miR-3162, miR-4299, miR-572, miR-575, miR-630, miR-642b: ↓	5 μM for 24 h	Cell culture	H_2_O_2_-exposed HaCaT cells	/	● Protected against H_2_O_2_-induced oxidative stress.	[87]
Polydatin 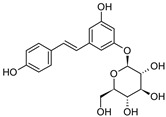	miR-200a↑	10, 20, and 40 μM for 24 h; 7.5, 15, and 30 mg/kg/d for 7 weeks	Cell culture; gavage	High fructose-treated buffalo rat liver cells and HepG2 cells; fructose-fed rats	Keap1	● Alleviated hepatic oxidative stress, inflammation, and lipid deposition; ● Activated Keap1/Nrf2 pathway.	[88]
Sodium tanshinone IIA sulfonate 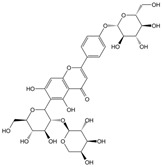	miR-133a↓	10 mg/kg/d for 3 weeks	Gavage	STZ-induced diabetic mice	/	● Improved perfusion recovery; ● Increased capillary densities; ● Decreased ROS level and increased GCH-1 protein level.	[89]
Vaccarin 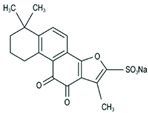	miR-34a↓	1 mg/kg/d for 4 weeks	Intraperitoneal injection	STZ/HFD-induced T2DM mice	/	● Reduced blood glucose; ● Increased glucose and insulin tolerance; ● Relieved the disorder of lipid metabolism and oxidative stress; ● Improved endothelium-dependent vasorelaxation.	[90]

The up arrow means an increase, and the down arrow means a decrease.

## Data Availability

Not applicable.

## Abbreviations

ABCA1: ATP-binding cassette transporter A1; ABCG1, adenosine triphosphate binding cassette transporter G1; ACC, acetyl-CoA carboxylase; ACLY, ATP citrate lyase; ALT, alanine aminotransferase; AMPK, AMP-activated protein kinase; aP2, adipocyte fatty acid binding protein; APOA5, apolipoprotein A5; AST, aspartate aminotransferase; Bcl-2, B cell lymphoma 2; C/EBPα, CCAAT/enhancer-binding protein alpha; CAT, catalase; Cav3, caveolin-3; Cols, collagens; CPT1a, carnitine palmitoyltransferase 1a; CTGF, connective tissue growth factor; CVB3, coxsackievirus B3; Cx43, connexin 43; Dvl-1, dishevelled 1; ECG, (-)-Epicatechin-3-gallate; EGCG, Epigallocatechin-3-gallate; ER, endoplasmic reticulum; ERK, extracellular signal-regulated kinases; Ets2, E26 oncogene homolog 2; FABP7, fatty acid-binding protein 7; FAS, fatty acid synthase; Fbn1, fibrillin 1; FOXO1, forkhead box O1; GCH-1, GTP cyclohydrolase-1; GSH-Px, glutathione peroxidase; GSK3β, glycogen synthase kinase 3β; HDL, high density lipoproteins; HDL-C, high density lipoproteins cholesterol; HFD, high-fat diet; HMGCS, 3-hydroxy-3-methylglutaryl-CoA synthase; HO-1, heme oxygenase-1; HUVECs, human umbilical vascular endothelial cells; I/R, ischemia reperfusion injury; INSIG2, insulin-induced gene 2; IR, insulin receptor; IRS1, insulin receptor substrate 1; ISO, isoproterenol; ITT, insulin tolerance test; KCNJ2, Kir2.1 mRNA; Keap1, Kelch-like ECH-associated protein 1; KLF4, Krüppel-like factor 4; LDL-C, low density lipoproteins cholesterol; LPL, lipoprotein lipase; MAPK7, mitogen-activated protein kinase 7; MCE, mitotic clonal expansion; MCL-1, myeloid cell leukemia 1; MDA, malondialdehyde; MET, hepatocyte growth factor receptor; NASH, nonalcoholic steatohepatitis; NPC1, Niemann-Pick C1; Nrf2, nuclear factor erythroid 2-related factor 2; OGG1, 8-oxoguanine-DNA glycosylase; oxLDL, low density lipoprotein; PAD, peripheral arterial disease; PDX1, pancreatic duodenal homeobox-1; PGC1α, peroxisome proliferator-activated receptor gamma coactivator-1alpha; PGC1β, peroxisome proliferator-activated receptor gamma coactivator-1beta; PPARβ/δ, peroxisome proliferator-activated receptor beta/delta; PPARγ, peroxisome proliferator-activated receptor-gamma; PTEN, phosphatase Phosphatase and tensin homologue deleted on chromosome 10; PTP1B, protein tyrosine phosphatase 1B; ROS, reactive oxygen species; SCD1, stearoyl-coenzyme A desaturase 1; Sirt1, sirtuin type 1; SOD, superoxide dismutase; SP1, Sp1 transcription factor; SREBP1c, sterol regulatory element binding protein 1c; SRF, serum response factor; STZ, streptozotocin; T2DM, type 2 diabetes mellitus; TC, total cholesterol; TCF7L2, transcription factor 7-like 2; TG, triglyceride; TGF-β, transforming growth factor β; TRPM7, transient receptor potential melastatin 7; TRPV1, transient receptor potential cation channel subfamily V member 1; VCAM-1, vascular cell adhesionmolecule-1; VSMCs, vascular smooth muscle cells; Wnt1, wingless-type MMTV integration site family member 1; Wnt10b, wingless-type MMTV integration site family member 10b; Wnt3a, wingless-type MMTV integration site family member 3a; Wnt4, wingless-type MMTV integration site family member 4; ZEB2, zinc finger E-Box binding homeobox 2.

## References

[1] Cawley J., Wen K. (2018). Policies to Prevent Obesity and Promote Healthier Diets: A Critical Selective Review. Clin. Chem..

[2] Unnikrishnan R., Pradeepa R., Joshi S.R., Mohan V. (2017). Type 2 Diabetes: Demystifying the Global Epidemic. Diabetes.

[3] Kan J., Velliquette R.A., Grann K., Burns C.R., Scholten J., Tian F., Zhang Q., Gui M. (2017). A novel botanical formula prevents diabetes by improving insulin resistance. BMC Complement. Altern. Med..

[4] Smilowitz N.R., Gupta N., Guo Y., Beckman J.A., Bangalore S., Berger J.S. (2018). Trends in cardiovascular risk factor and disease prevalence in patients undergoing non-cardiac surgery. Heart.

[5] Derosa G., Maffioli P. (2012). Anti-obesity drugs: A review about their effects and their safety. Expert Opin. Drug Saf..

[6] Kang J.G., Park C.Y. (2012). Anti-Obesity Drugs: A Review about Their Effects and Safety. Diabetes Metab. J..

[7] Dietrich M.O., Horvath T.L. (2012). Limitations in anti-obesity drug development: The critical role of hunger-promoting neurons. Nat. Rev. Drug Discov..

[8] Baker D.D., Chu M., Oza U., Rajgarhia V. (2007). The value of natural products to future pharmaceutical discovery. Nat. Prod. Rep..

[9] Koehn F.E., Carter G.T. (2005). The evolving role of natural products in drug discovery. Nat. Rev. Drug Discov..

[10] Martel J., Ojcius D.M., Chang C.J., Lin C.S., Lu C.C., Ko Y.F., Tseng S.F., Lai H.C., Young J.D. (2017). Anti-obesogenic and antidiabetic effects of plants and mushrooms. Nat. Rev. Endocrinol..

[11] Shukla S.K., Gupta S., Ojha S.K., Sharma S.B. (2010). Cardiovascular friendly natural products: A promising approach in the management of CVD. Nat. Prod. Res..

[12] Cragg G.M., Newman D.J. (2013). Natural products: A continuing source of novel drug leads. Biochim. Biophys. Acta.

[13] Yao H., Liu J., Xu S., Zhu Z., Xu J. (2017). The structural modification of natural products for novel drug discovery. Expert Opin. Drug Discov..

[14] Martello G., Rosato A., Ferrari F., Manfrin A., Cordenonsi M., Dupont S., Enzo E., Guzzardo V., Rondina M., Spruce T. (2010). A MicroRNA targeting dicer for metastasis control. Cell.

[15] Bartel D.P. (2004). MicroRNAs: Genomics, biogenesis, mechanism, and function. Cell.

[16] Baselga-Escudero L., Pascual-Serrano A., Ribas-Latre A., Casanova E., Salvado M.J., Arola L., Arola-Arnal A., Blade C. (2015). Long-term supplementation with a low dose of proanthocyanidins normalized liver miR-33a and miR-122 levels in high-fat diet-induced obese rats. Nutr. Res..

[17] Zhang L., He S., Yang F., Yu H., Xie W., Dai Q., Zhang D., Liu X., Zhou S., Zhang K. (2016). Hyperoside ameliorates glomerulosclerosis in diabetic nephropathy by downregulating miR-21. Can. J. Physiol. Pharmacol..

[18] Liu L., Ning B., Cui J., Zhang T., Chen Y. (2017). miR-29c is implicated in the cardioprotective activity of Panax notoginseng saponins against isoproterenol-induced myocardial fibrogenesis. J. Ethnopharmacol..

[19] Tung Y.T., Chen H.L., Wu H.S., Ho M.H., Chong K.Y., Chen C.M. (2017). Kefir Peptides Prevent Hyperlipidemia and Obesity in High-Fat-Diet-Induced Obese Rats via Lipid Metabolism Modulation. Mol. Nutr. Food Res..

[20] Pang D., You L., Zhou L., Li T., Zheng B., Liu R.H. (2017). Averrhoa carambola free phenolic extract ameliorates nonalcoholic hepatic steatosis by modulating mircoRNA-34a, mircoRNA-33 and AMPK pathways in leptin receptor-deficient db/db mice. Food Funct..

[21] Liu S., Chang X., Yu J., Xu W. (2020). Cerasus humilis Cherry Polyphenol Reduces High-Fat Diet-Induced Obesity in C57BL/6 Mice by Mitigating Fat Deposition, Inflammation, and Oxidation. J. Agric. Food Chem..

[22] Su D., Liu H., Qi X., Dong L., Zhang R., Zhang J. (2019). Citrus peel flavonoids improve lipid metabolism by inhibiting miR-33 and miR-122 expression in HepG2 cells. Biosci. Biotechnol. Biochem..

[23] Murase T., Misawa K., Minegishi Y., Aoki M., Ominami H., Suzuki Y., Shibuya Y., Hase T. (2011). Coffee polyphenols suppress diet-induced body fat accumulation by downregulating SREBP-1c and related molecules in C57BL/6J mice. Am. J. Physiol. Endocrinol. Metab..

[24] Kim S., Lee M.S., Jung S., Son H.Y., Park S., Kang B., Kim S.Y., Kim I.H., Kim C.T., Kim Y. (2018). Ginger Extract Ameliorates Obesity and Inflammation via Regulating MicroRNA-21/132 Expression and AMPK Activation in White Adipose Tissue. Nutrients.

[25] Baselga-Escudero L., Blade C., Ribas-Latre A., Casanova E., Salvado M.J., Arola L., Arola-Arnal A. (2014). Chronic supplementation of proanthocyanidins reduces postprandial lipemia and liver miR-33a and miR-122 levels in a dose-dependent manner in healthy rats. J. Nutr. Biochem..

[26] Baselga-Escudero L., Blade C., Ribas-Latre A., Casanova E., Salvado M.J., Arola L., Arola-Arnal A. (2012). Grape seed proanthocyanidins repress the hepatic lipid regulators miR-33 and miR-122 in rats. Mol. Nutr. Food Res..

[27] Baselga-Escudero L., Arola-Arnal A., Pascual-Serrano A., Ribas-Latre A., Casanova E., Salvado M.J., Arola L., Blade C. (2013). Chronic administration of proanthocyanidins or docosahexaenoic acid reverses the increase of miR-33a and miR-122 in dyslipidemic obese rats. PLoS ONE.

[28] Shi Y., Jia M., Xu L., Fang Z., Wu W., Zhang Q., Chung P., Lin Y., Wang S., Zhang Y. (2019). miR-96 and autophagy are involved in the beneficial effect of grape seed proanthocyanidins against high-fat-diet-induced dyslipidemia in mice. Phytother. Res..

[29] Lu R.H., Qin C.B., Yang F., Zhang W.Y., Zhang Y.R., Yang G.K., Yang L.P., Meng X.L., Yan X., Nie G.X. (2020). Grape seed proanthocyanidin extract ameliorates hepatic lipid accumulation and inflammation in grass carp (*Ctenopharyngodon idella*). Fish Physiol. Biochem..

[30] Torres L.F., Cogliati B., Otton R. (2019). Green Tea Prevents NAFLD by Modulation of miR-34a and miR-194 Expression in a High-Fat Diet Mouse Model. Oxid. Med. Cell. Longev..

[31] Otton R., Bolin A.P., Ferreira L.T., Marinovic M.P., Rocha A.L.S., Mori M.A. (2018). Polyphenol-rich green tea extract improves adipose tissue metabolism by down-regulating miR-335 expression and mitigating insulin resistance and inflammation. J. Nutr. Biochem..

[32] Lima N.D.S., Numata E.P., Mesquita L.M.S., Dias P.H., Vilegas W., Gambero A., Ribeiro M.L. (2017). Modulatory Effects of Guarana (*Paullinia cupana*) on Adipogenesis. Nutrients.

[33] Su D., Zhang R., Hou F., Chi J., Huang F., Yan S., Liu L., Deng Y., Wei Z., Zhang M. (2017). Lychee pulp phenolics ameliorate hepatic lipid accumulation by reducing miR-33 and miR-122 expression in mice fed a high-fat diet. Food Funct..

[34] Lee S., Lee M.S., Chang E., Lee Y., Lee J., Kim J., Kim C.T., Kim I.H., Kim Y. (2020). Mulberry Fruit Extract Promotes Serum HDL-Cholesterol Levels and Suppresses Hepatic microRNA-33 Expression in Rats Fed High Cholesterol/Cholic Acid Diet. Nutrients.

[35] Yang T.Y., Yu M.H., Wu Y.L., Hong C.C., Chen C.S., Chan K.C., Wang C.J. (2022). Mulberry Leaf (*Morus alba* L.) Extracts and Its Chlorogenic Acid Isomer Component Improve Glucolipotoxicity-Induced Hepatic Lipid Accumulation via Downregulating miR-34a and Decreased Inflammation. Nutrients.

[36] Monraz-Méndez C.A., Escutia-Gutiérrez R., Rodriguez-Sanabria J.S., Galicia-Moreno M., Monroy-Ramírez H.C., Sánchez-Orozco L., García-Bañuelos J., de la Rosa-Bibiano R., Santos A., Armendáriz-Borunda J. (2022). Moringa oleifera Improves MAFLD by Inducing Epigenetic Modifications. Nutrients.

[37] Qiao J.Y., Li H.W., Liu F.G., Li Y.C., Tian S., Cao L.H., Hu K., Wu X.X., Miao M.S. (2019). Effects of *Portulaca Oleracea* Extract on Acute Alcoholic Liver Injury of Rats. Molecules.

[38] Jang S., Lee M.S., Kang S.A., Kim C.T., Kim Y. (2022). *Portulaca oleracea* L. Extract Regulates Hepatic Cholesterol Metabolism via the AMPK/MicroRNA-33/34a Pathway in Rats Fed a High-Cholesterol Diet. Nutrients.

[39] Stefanon B., Pomari E., Colitti M. (2015). Effects of Rosmarinus officinalis extract on human primary omental preadipocytes and adipocytes. Exp. Biol. Med..

[40] Zhu W., Zou B., Nie R., Zhang Y., Li C.M. (2015). A-type ECG and EGCG dimers disturb the structure of 3T3-L1 cell membrane and strongly inhibit its differentiation by targeting peroxisome proliferator-activated receptor gamma with miR-27 involved mechanism. J. Nutr. Biochem..

[41] Tian L., Song Z., Shao W., Du W.W., Zhao L.R., Zeng K., Yang B.B., Jin T. (2017). Curcumin represses mouse 3T3-L1 cell adipogenic differentiation via inhibiting miR-17-5p and stimulating the Wnt signalling pathway effector Tcf7l2. Cell Death Dis..

[42] Zhang J., Huang Y., Shao H., Bi Q., Chen J., Ye Z. (2017). Grape seed procyanidin B2 inhibits adipogenesis of 3T3-L1 cells by targeting peroxisome proliferator-activated receptor gamma with miR-483-5p involved mechanism. Biomed. Pharmacother..

[43] Chen C.P., Su T.C., Yang M.J., Chen W.T., Siao A.C., Huang L.R., Lin Y.Y., Kuo Y.C., Chung J.F., Cheng C.F. (2022). Green tea epigallocatechin gallate suppresses 3T3-L1 cell growth via microRNA-143/MAPK7 pathways. Exp. Biol. Med..

[44] Ahn J., Lee H., Jung C.H., Ha T. (2012). Lycopene inhibits hepatic steatosis via microRNA-21-induced downregulation of fatty acid-binding protein 7 in mice fed a high-fat diet. Mol. Nutr. Food Res..

[45] Rohm B., Holik A.K., Kretschy N., Somoza M.M., Ley J.P., Widder S., Krammer G.E., Marko D., Somoza V. (2015). Nonivamide enhances miRNA let-7d expression and decreases adipogenesis PPARgamma expression in 3T3-L1 cells. J. Cell. Biochem..

[46] Chen S., Wen X., Zhang W., Wang C., Liu J., Liu C. (2017). Hypolipidemic effect of oleanolic acid is mediated by the miR-98-5p/PGC-1beta axis in high-fat diet-induced hyperlipidemic mice. FASEB J..

[47] Zou B., Ge Z., Zhu W., Xu Z., Li C. (2015). Persimmon tannin represses 3T3-L1 preadipocyte differentiation via up-regulating expression of miR-27 and down-regulating expression of peroxisome proliferator-activated receptor-gamma in the early phase of adipogenesis. Eur. J. Nutr..

[48] Gai Y., Li Y., Xu Z., Chen J. (2019). Pseudoprotodioscin inhibits SREBPs and microRNA 33a/b levels and reduces the gene expression regarding the synthesis of cholesterol and triglycerides. Fitoterapia.

[49] Gracia A., Fernandez-Quintela A., Miranda J., Eseberri I., Gonzalez M., Portillo M.P. (2017). Are miRNA-103, miRNA-107 and miRNA-122 Involved in the Prevention of Liver Steatosis Induced by Resveratrol?. Nutrients.

[50] Gracia A., Miranda J., Fernandez-Quintela A., Eseberri I., Garcia-Lacarte M., Milagro F.I., Martinez J.A., Aguirre L., Portillo M.P. (2016). Involvement of miR-539-5p in the inhibition of de novo lipogenesis induced by resveratrol in white adipose tissue. Food Funct..

[51] Eseberri I., Lasa A., Miranda J., Gracia A., Portillo M.P. (2017). Potential miRNA involvement in the anti-adipogenic effect of resveratrol and its metabolites. PLoS ONE.

[52] Ahn J., Lee H., Jung C.H., Choi W.H., Ha T.Y. (2017). Zerumbone ameliorates high-fat diet-induced adiposity by restoring AMPK-regulated lipogenesis and microRNA-146b/SIRT1-mediated adipogenesis. Oncotarget.

[53] Rottiers V., Naar A.M. (2012). MicroRNAs in metabolism and metabolic disorders. Nat. Rev. Mol. Cell Biol..

[54] Moore K.J., Rayner K.J., Suarez Y., Fernandez-Hernando C. (2011). The role of microRNAs in cholesterol efflux and hepatic lipid metabolism. Annu. Rev. Nutr..

[55] Qiu C.P., Lv Q.T., Dongol S., Wang C., Jiang J. (2014). Single nucleotide polymorphism of SREBF-1 gene associated with an increased risk of endometrial cancer in Chinese women. PLoS ONE.

[56] Horton J.D., Goldstein J.L., Brown M.S. (2002). SREBPs: Activators of the complete program of cholesterol and fatty acid synthesis in the liver. J. Clin. Investig..

[57] Nagai Y., Yonemitsu S., Erion D.M., Iwasaki T., Stark R., Weismann D., Dong J., Zhang D., Jurczak M.J., Loffler M.G. (2009). The role of peroxisome proliferator-activated receptor gamma coactivator-1 beta in the pathogenesis of fructose-induced insulin resistance. Cell Metab..

[58] Bennett M.K., Lopez J.M., Sanchez H.B., Osborne T.F. (1995). Sterol regulation of fatty acid synthase promoter. Coordinate feedback regulation of two major lipid pathways. J. Biol. Chem..

[59] Sullivan J.E., Brocklehurst K.J., Marley A.E., Carey F., Carling D., Beri R.K. (1994). Inhibition of lipolysis and lipogenesis in isolated rat adipocytes with AICAR, a cell-permeable activator of AMP-activated protein kinase. FEBS Lett..

[60] Daval M., Foufelle F., Ferre P. (2006). Functions of AMP-activated protein kinase in adipose tissue. J. Physiol..

[61] Saha A.K., Schwarsin A.J., Roduit R., Masse F., Kaushik V., Tornheim K., Prentki M., Ruderman N.B. (2000). Activation of malonyl-CoA decarboxylase in rat skeletal muscle by contraction and the AMP-activated protein kinase activator 5-aminoimidazole-4-carboxamide-1-beta-D-ribofuranoside. J. Biol. Chem..

[62] Bengestrate L., Virtue S., Campbell M., Vidal-Puig A., Hadaschik D., Hahn P., Bielke W. (2011). Genome-wide profiling of microRNAs in adipose mesenchymal stem cell differentiation and mouse models of obesity. PLoS ONE.

[63] Tang Q.Q., Lane M.D. (2012). Adipogenesis: From stem cell to adipocyte. Annu. Rev. Biochem..

[64] Gregoire F.M., Smas C.M., Sul H.S. (1998). Understanding adipocyte differentiation. Physiol. Rev..

[65] Nickels J.D., Chatterjee S., Stanley C.B., Qian S., Cheng X., Myles D.A.A., Standaert R.F., Elkins J.G., Katsaras J. (2017). The in vivo structure of biological membranes and evidence for lipid domains. PLoS Biol..

[66] Rosen E.D., MacDougald O.A. (2006). Adipocyte differentiation from the inside out. Nat. Rev. Mol. Cell Biol..

[67] Price N.L., Fernandez-Hernando C. (2016). miRNA regulation of white and brown adipose tissue differentiation and function. Biochim. Biophys. Acta.

[68] Chen Q., Shou P., Zheng C., Jiang M., Cao G., Yang Q., Cao J., Xie N., Velletri T., Zhang X. (2016). Fate decision of mesenchymal stem cells: Adipocytes or osteoblasts?. Cell Death Differ..

[69] Ross S.E., Hemati N., Longo K.A., Bennett C.N., Lucas P.C., Erickson R.L., MacDougald O.A. (2000). Inhibition of adipogenesis by Wnt signaling. Science.

[70] Dogan A., Demirci S., Apdik H., Bayrak O.F., Gulluoglu S., Tuysuz E.C., Gusev O., Rizvanov A.A., Nikerel E., Sahin F. (2017). A new hope for obesity management: Boron inhibits adipogenesis in progenitor cells through the Wnt/beta-catenin pathway. Metabolism.

[71] Christodoulides C., Lagathu C., Sethi J.K., Vidal-Puig A. (2009). Adipogenesis and WNT signalling. Trends Endocrinol. Metab..

[72] Cuadros D.F., Li J., Musuka G., Awad S.F. (2021). Spatial epidemiology of diabetes: Methods and insights. World J. Diabetes.

[73] Cho N.H., Shaw J.E., Karuranga S., Huang Y., da Rocha Fernandes J.D., Ohlrogge A.W., Malanda B. (2018). IDF Diabetes Atlas: Global estimates of diabetes prevalence for 2017 and projections for 2045. Diabetes Res. Clin. Pract..

[74] Alam U., Asghar O., Azmi S., Malik R.A. (2014). General aspects of diabetes mellitus. Handb. Clin. Neurol..

[75] Kim A., Lee W., Yun J.M. (2017). Luteolin and fisetin suppress oxidative stress by modulating sirtuins and forkhead box O3a expression under in vitro diabetic conditions. Nutr. Res. Pract..

[76] Du G., Xiao M., Zhang X., Wen M., Pang C., Jiang S., Sang S., Xie Y. (2017). Alpinia oxyphylla Miq. extract changes miRNA expression profiles in db-/db- mouse kidney. Biol. Res..

[77] Wang C., Wang K., Li P. (2022). Blueberry anthocyanins extract attenuated diabetic retinopathy by inhibiting endoplasmic reticulum stress via the miR-182/OGG1 axis. J. Pharmacol. Sci..

[78] Yu S., Zhao H., Yang W., Amat R., Peng J., Li Y., Deng K., Mao X., Jiao Y. (2019). The Alcohol Extract of Coreopsis tinctoria Nutt Ameliorates Diabetes and Diabetic Nephropathy in db/db Mice through miR-192/miR-200b and PTEN/AKT and ZEB2/ECM Pathways. Biomed. Res. Int..

[79] Asgari M., Salehi I., Ranjbar K., Khosravi M., Zarrinkalam E. (2022). Interval training and Crataegus persica ameliorate diabetic nephropathy via miR-126/Nrf-2 mediated inhibition of stress oxidative in rats with diabetes after myocardial ischemia-reperfusion injury. Biomed. Pharmacother..

[80] Yang Y.M., Seo S.Y., Kim T.H., Kim S.G. (2012). Decrease of microRNA-122 causes hepatic insulin resistance by inducing protein tyrosine phosphatase 1B, which is reversed by licorice flavonoid. Hepatology.

[81] Balbaa M., Abdulmalek S.A., Khalil S. (2017). Oxidative stress and expression of insulin signaling proteins in the brain of diabetic rats: Role of Nigella sativa oil and antidiabetic drugs. PLoS ONE.

[82] Tang X., Li X., Zhang D., Han W. (2022). Astragaloside-IV alleviates high glucose-induced ferroptosis in retinal pigment epithelial cells by disrupting the expression of miR-138-5p/Sirt1/Nrf2. Bioengineered.

[83] Ding X.Q., Gu T.T., Wang W., Song L., Chen T.Y., Xue Q.C., Zhou F., Li J.M., Kong L.D. (2015). Curcumin protects against fructose-induced podocyte insulin signaling impairment through upregulation of miR-206. Mol. Nutr. Food Res..

[84] Zhang Y.M., Tao X.F., Yin L.H., Xu L., Xu Y.W., Qi Y., Han X., Song S.S., Zhao Y.Y., Lin Y. (2017). Protective effects of dioscin against cisplatin-induced nephrotoxicity via the microRNA-34a/sirtuin 1 signalling pathway. Br. J. Pharmacol..

[85] Zhang H., Zhao Z., Pang X., Yang J., Yu H., Zhang Y., Zhou H., Zhao J. (2017). MiR-34a/sirtuin-1/foxo3a is involved in genistein protecting against ox-LDL-induced oxidative damage in HUVECs. Toxicol. Lett..

[86] Li Y. (2022). Gypenoside A attenuates dysfunction of pancreatic β cells by activating PDX1 signal transduction via the inhibition of miR-150-3p both in vivo and in vitro. J. Biochem. Mol. Toxicol..

[87] Bae S., Lee E.J., Lee J.H., Park I.C., Lee S.J., Hahn H.J., Ahn K.J., An S., An I.S., Cha H.J. (2014). Oridonin protects HaCaT keratinocytes against hydrogen peroxide-induced oxidative stress by altering microRNA expression. Int. J. Mol. Med..

[88] Zhao X.J., Yu H.W., Yang Y.Z., Wu W.Y., Chen T.Y., Jia K.K., Kang L.L., Jiao R.Q., Kong L.D. (2018). Polydatin prevents fructose-induced liver inflammation and lipid deposition through increasing miR-200a to regulate Keap1/Nrf2 pathway. Redox Biol..

[89] Chen L., He W., Peng B., Yuan M., Wang N., Wang J., Lu W., Wang T. (2019). Sodium Tanshinone IIA sulfonate improves post-ischemic angiogenesis in hyperglycemia. Biochem. Biophys. Res. Commun..

[90] Xu F., Liu Y., Zhu X., Li S., Shi X., Li Z., Ai M., Sun J., Hou B., Cai W. (2019). Protective Effects and Mechanisms of Vaccarin on Vascular Endothelial Dysfunction in Diabetic Angiopathy. Int. J. Mol. Sci..

[91] Azimova K., Rude J., Mallawaarachchi I., Dwivedi A., Sarosiek J., Mukherjee D. (2015). Glucose levels and depression in Hispanic patients admitted to the cardiovascular intensive care unit: A cross-sectional study. Angiology.

[92] Stanisic J., Koricanac G., Culafic T., Romic S., Stojiljkovic M., Kostic M., Pantelic M., Tepavcevic S. (2016). Low intensity exercise prevents disturbances in rat cardiac insulin signaling and endothelial nitric oxide synthase induced by high fructose diet. Mol. Cell. Endocrinol..

[93] Goldstein B.J. (1993). Regulation of insulin receptor signaling by protein-tyrosine dephosphorylation. Receptor.

[94] Evans J.L., Goldfine I.D., Maddux B.A., Grodsky G.M. (2003). Are oxidative stress-activated signaling pathways mediators of insulin resistance and beta-cell dysfunction?. Diabetes.

[95] Mao X., Gu C., Chen D., Yu B., He J. (2017). Oxidative stress-induced diseases and tea polyphenols. Oncotarget.

[96] Keane K.N., Cruzat V.F., Carlessi R., de Bittencourt P.I.H., Newsholme P. (2015). Molecular Events Linking Oxidative Stress and Inflammation to Insulin Resistance and beta-Cell Dysfunction. Oxid. Med. Cell. Longev..

[97] Guo Q., Xu L., Li H., Sun H., Liu J., Wu S., Zhou B. (2017). Progranulin causes adipose insulin resistance via increased autophagy resulting from activated oxidative stress and endoplasmic reticulum stress. Lipids Health Dis..

[98] Ma L., Li Y. (2015). SIRT1: Role in cardiovascular biology. Clin. Chim. Acta.

[99] Jamal J., Mustafa M.R., Wong P.F. (2014). Paeonol protects against premature senescence in endothelial cells by modulating Sirtuin 1 pathway. J. Ethnopharmacol..

[100] Do M.T., Kim H.G., Choi J.H., Jeong H.G. (2014). Metformin induces microRNA-34a to downregulate the Sirt1/Pgc-1alpha/Nrf2 pathway, leading to increased susceptibility of wild-type p53 cancer cells to oxidative stress and therapeutic agents. Free Radic. Biol. Med..

[101] Yin L.H., Xu Y.W., Qi Y., Han X., Xu L.N., Peng J.Y., Sun C.K. (2010). A green and efficient protocol for industrial-scale preparation of dioscin from Dioscorea nipponica Makino by two-step macroporous resin column chromatography. Chem. Eng. J..

[102] Chen W., Gao R., Liu L., Zhu M., Wang W., Wang Y., Wu Z., Li H., Zheng Z., Jiang L. (2016). Outline of the report on cardiovascular diseases in China, 2014. Eur. Heart J. Suppl..

[103] Le Bouc Y., Brioude F. (2012). Is there a relationship between the growth hormone dose and tumoral or cardiovascular complications?. Bull. Acad. Natl. Med..

[104] Sadowska J., Bruszkowska M. (2017). Comparing the effects of sucrose and high-fructose corn syrup on lipid metabolism and the risk of cardiovascular disease in male rats. Acta Sci. Pol. Technol. Aliment..

[105] Traverse J.H., Henry T.D. (2017). Myocardial Injury as a New Target for Cell Therapy in Patients with Chronic Heart Failure When Something Bad Is Actually Good?. Circ. Res..

[106] Liu H., Jing X., Dong A., Bai B., Wang H. (2017). Overexpression of TIMP3 Protects Against Cardiac Ischemia/Reperfusion Injury by Inhibiting Myocardial Apoptosis through ROS/Mapks Pathway. Cell. Physiol. Biochem..

[107] Zhang X., Pan L., Yang K., Fu Y., Liu Y., Chi J., Zhang X., Hong S., Ma X., Yin X. (2017). H3 Relaxin Protects Against Myocardial Injury in Experimental Diabetic Cardiomyopathy by Inhibiting Myocardial Apoptosis, Fibrosis and Inflammation. Cell. Physiol. Biochem..

[108] Li F., Zong J., Zhang H., Zhang P., Xu L., Liang K., Yang L., Yong H., Qian W. (2017). Orientin Reduces Myocardial Infarction Size via eNOS/NO Signaling and thus Mitigates Adverse Cardiac Remodeling. Front. Pharmacol..

[109] Zhang Y., Zhang L., Chu W.F., Wang B., Zhang J.L., Zhao M., Li X.L., Li B.X., Lu Y.J., Yang B.F. (2010). Tanshinone IIA Inhibits miR-1 Expression through p38 MAPK Signal Pathway in Post-infarction Rat Cardiomyocytes. Cell. Physiol. Biochem..

[110] Zhang L., Wu Y., Li Y., Xu C., Li X., Zhu D., Zhang Y., Xing S., Wang H., Zhang Z. (2012). Tanshinone IIA improves miR-133 expression through MAPK ERK1/2 pathway in hypoxic cardiac myocytes. Cell. Physiol. Biochem..

[111] Shan H., Li X., Pan Z., Zhang L., Cai B., Zhang Y., Xu C., Chu W., Qiao G., Li B. (2009). Tanshinone IIA protects against sudden cardiac death induced by lethal arrhythmias via repression of microRNA-1. Br. J. Pharmacol..

[112] Miano J.M., Ramanan N., Georger M.A., de Mesy Bentley K.L., Emerson R.L., Balza R.O., Xiao Q., Weiler H., Ginty D.D., Misra R.P. (2004). Restricted inactivation of serum response factor to the cardiovascular system. Proc. Natl. Acad. Sci. USA.

[113] Yang B., Ma S., Wang Y.B., Xu B., Zhao H., He Y.Y., Li C.W., Zhang J., Cao Y.K., Feng Q.Z. (2016). Resveratrol exerts protective effects on anoxia/reoxygenation injury in cardiomyocytes via miR-34a/Sirt1 signaling pathway. Eur. Rev. Med. Pharmacol. Sci..

[114] Chang L., Shi R., Wang X., Bao Y. (2020). Gypenoside A protects ischemia/reperfusion injuries by suppressing miR-143-3p level via the activation of AMPK/Foxo1 pathway. Biofactors.

[115] Hussein R.M., Youssef A.M., Magharbeh M.K., Al-Dalaen S.M., Al-Jawabri N.A., Al-Nawaiseh T.N., Al-Jwanieh A., Al-Ani F.S. (2022). Protective Effect of *Portulaca oleracea* Extract Against Lipopolysaccharide-Induced Neuroinflammation, Memory Decline, and Oxidative Stress in Mice: Potential Role of miR-146a and miR-let 7. J. Med. Food.

[116] Zhao L., Tao X., Qi Y., Xu L., Yin L., Peng J. (2018). Protective effect of dioscin against doxorubicin-induced cardiotoxicity via adjusting microRNA-140-5p-mediated myocardial oxidative stress. Redox Biol..

[117] Qin F., Siwik D.A., Pimentel D.R., Morgan R.J., Biolo A., Tu V.H., Kang Y.J., Cohen R.A., Colucci W.S. (2014). Cytosolic H_2_O_2_ mediates hypertrophy, apoptosis, and decreased SERCA activity in mice with chronic hemodynamic overload. Am. J. Physiol. Heart Circ. Physiol..

[118] Yang T.R., Zhang T., Mu N.H., Ruan L.B., Duan J.L., Zhang R.P., Miao Y.B. (2019). Resina draconis inhibits the endoplasmic-reticulum-induced apoptosis of myocardial cells via regulating miR-423-3p/ERK signaling pathway in a tree shrew myocardial ischemia- reperfusion model. J. Biosci..

[119] Lin J., Zheng X. (2017). Salvianolate Blocks Apoptosis During Myocardial Infarction by Down Regulating miR-122-5p. Curr. Neurovasc. Res..

[120] Yin K., Zhao L., Feng D., Ma W., Liu Y., Wang Y., Liang J., Yang F., Bi C., Chen H. (2015). Resveratrol Attenuated Low Ambient Temperature-Induced Myocardial Hypertrophy via Inhibiting Cardiomyocyte Apoptosis. Cell. Physiol. Biochem..

[121] Lee B.S., Oh J., Kang S.K., Park S., Lee S.H., Choi D., Chung J.H., Chung Y.W., Kang S.M. (2015). Insulin Protects Cardiac Myocytes from Doxorubicin Toxicity by Sp1-Mediated Transactivation of Survivin. PLoS ONE.

[122] Geng H.H., Li R., Su Y.M., Xiao J., Pan M., Cai X.X., Ji X.P. (2016). Curcumin protects cardiac myocyte against hypoxia-induced apoptosis through upregulating miR-7a/b expression. Biomed. Pharmacother..

[123] Wang L., Cui Y., Tang M., Hu X., Luo H., Hescheler J., Xi J. (2014). Puerarin facilitates T-tubule development of murine embryonic stem cell-derived cardiomyocytes. Cell. Physiol. Biochem..

[124] Yan X., Liu J., Wu H., Liu Y., Zheng S., Zhang C., Yang C. (2016). Impact of miR-208 and its Target Gene Nemo-Like Kinase on the Protective Effect of Ginsenoside Rb1 in Hypoxia/Ischemia Injuried Cardiomyocytes. Cell. Physiol. Biochem..

[125] Zeng J., Deng Z., Zou Y., Liu C., Fu H., Gu Y., Chang H. (2021). Theaflavin alleviates oxidative injury and atherosclerosis progress via activating microRNA-24-mediated Nrf2/HO-1 signal. Phytother. Res..

[126] Wang Y., Li J., Xuan L., Liu Y., Shao L., Ge H., Gu J., Wei C., Zhao M. (2018). Astragalus Root dry extract restores connexin43 expression by targeting miR-1 in viral myocarditis. Phytomedicine.

[127] Li X., Zhao D., Guo Z., Li T., Qili M., Xu B., Qian M., Liang H., E X., Chege Gitau S. (2016). Overexpression of SerpinE2/protease nexin-1 Contribute to Pathological Cardiac Fibrosis via increasing Collagen Deposition. Sci. Rep..

[128] Kannaiyan R., Shanmugam M.K., Sethi G. (2011). Molecular targets of celastrol derived from Thunder of God Vine: Potential role in the treatment of inflammatory disorders and cancer. Cancer Lett..

[129] Cheng M., Wu G., Song Y., Wang L., Tu L., Zhang L., Zhang C. (2016). Celastrol-Induced Suppression of the MiR-21/ERK Signalling Pathway Attenuates Cardiac Fibrosis and Dysfunction. Cell. Physiol. Biochem..

[130] Ning B.B., Zhang Y., Wu D.D., Cui J.G., Liu L., Wang P.W., Wang W.J., Zhu W.L., Chen Y., Zhang T. (2017). Luteolin-7-diglucuronide attenuates isoproterenol-induced myocardial injury and fibrosis in mice. Acta Pharmacol. Sin..

[131] Wei Y., Wu Y., Feng K., Zhao Y., Tao R., Xu H., Tang Y. (2020). Astragaloside IV inhibits cardiac fibrosis via miR-135a-TRPM7-TGF-β/Smads pathway. J. Ethnopharmacol..

[132] Noratto G.D., Angel-Morales G., Talcott S.T., Mertens-Talcott S.U. (2011). Polyphenolics from acai (*Euterpe oleracea* Mart.) and red muscadine grape (*Vitis rotundifolia*) protect human umbilical vascular Endothelial cells (HUVEC) from glucose- and lipopolysaccharide (LPS)-induced inflammation and target microRNA-126. J. Agric. Food Chem..

[133] Nie W., Zhang X., Yan H., Li S., Zhu W., Fan F., Zhu J. (2016). Xiaoxianggou attenuates atherosclerotic plaque formation in endogenous high Ang II ApoE(−/−) mice via the inhibition of miR-203 on the expression of Ets-2 in endothelial cells. Biomed. Pharmacother..

[134] Yin S., Bai W., Li P., Jian X., Shan T., Tang Z., Jing X., Ping S., Li Q., Miao Z. (2019). Berberine suppresses the ectopic expression of miR-133a in endothelial cells to improve vascular dementia in diabetic rats. Clin. Exp. Hypertens..

[135] Hua J.Y., He Y.Z., Xu Y., Jiang X.H., Ye W., Pan Z.M. (2015). Emodin prevents intima thickness via Wnt4/Dvl-1/beta-catenin signaling pathway mediated by miR-126 in balloon-injured carotid artery rats. Exp. Mol. Med..

[136] Kwok H.H., Chan L.S., Poon P.Y., Yue P.Y., Wong R.N. (2015). Ginsenoside-Rg1 induces angiogenesis by the inverse regulation of MET tyrosine kinase receptor expression through miR-23a. Toxicol. Appl. Pharmacol..

[137] Hibender S., Franken R., van Roomen C., Ter Braake A., van der Made I., Schermer E.E., Gunst Q., van den Hoff M.J., Lutgens E., Pinto Y.M. (2016). Resveratrol Inhibits Aortic Root Dilatation in the Fbn1C1039G/+ Marfan Mouse Model. Arterioscler. Thromb. Vasc. Biol..

[138] Chen W., Li X., Guo S., Song N., Wang J., Jia L., Zhu A. (2019). Tanshinone IIA harmonizes the crosstalk of autophagy and polarization in macrophages via miR-375/KLF4 pathway to attenuate atherosclerosis. Int. Immunopharmacol..

[139] Wang X.L., Fu A., Spiro C., Lee H.C. (2009). Proteomic Analysis of Vascular Endothelial Cells-Effects of Laminar Shear Stress and High Glucose. J. Proteom. Bioinform..

[140] Incalza M.A., D’Oria R., Natalicchio A., Perrini S., Laviola L., Giorgino F. (2017). Oxidative stress and reactive oxygen species in endothelial dysfunction associated with cardiovascular and metabolic diseases. Vascul. Pharmacol..

[141] Eren E., Yilmaz N., Aydin O. (2013). Functionally defective high-density lipoprotein and paraoxonase: A couple for endothelial dysfunction in atherosclerosis. Cholesterol.

[142] Dong Q., Xing W., Su F., Liang X., Tian F., Gao F., Wang S., Zhang H. (2017). Tetrahydroxystilbene Glycoside Improves Microvascular Endothelial Dysfunction and Ameliorates Obesity-Associated Hypertension in Obese ZDF Rats Via Inhibition of Endothelial Autophagy. Cell. Physiol. Biochem..

[143] Wilkinson E.L., Sidaway J.E., Cross M.J. (2018). Statin regulated ERK5 stimulates tight junction formation and reduces permeability in human cardiac endothelial cells. J. Cell. Physiol..

[144] Cai X., Bao L., Ding Y., Dai X., Zhang Z., Li Y. (2017). Quercetin alleviates cell apoptosis and inflammation via the ER stress pathway in vascular endothelial cells cultured in high concentrations of glucosamine. Mol. Med. Rep..

[145] Cheng C., Tempel D., den Dekker W.K., Haasdijk R., Chrifi I., Bos F.L., Wagtmans K., van de Kamp E.H., Blonden L., Biessen E.A. (2011). Ets2 determines the inflammatory state of endothelial cells in advanced atherosclerotic lesions. Circ. Res..

[146] Tsaousi A., Williams H., Lyon C.A., Taylor V., Swain A., Johnson J.L., George S.J. (2011). Wnt4/beta-catenin signaling induces VSMC proliferation and is associated with intimal thickening. Circ. Res..

[147] Chen Y., Lüttmann F.F., Schoger E., Schöler H.R., Zelarayán L.C., Kim K.P., Haigh J.J., Kim J., Braun T. (2021). Reversible reprogramming of cardiomyocytes to a fetal state drives heart regeneration in mice. Science.

[148] Cowan C.E., Kohler E.E., Dugan T.A., Mirza M.K., Malik A.B., Wary K.K. (2010). Kruppel-like factor-4 transcriptionally regulates VE-cadherin expression and endothelial barrier function. Circ. Res..

[149] Hale A.T., Tian H., Anih E., Recio F.O., Shatat M.A., Johnson T., Liao X., Ramirez-Bergeron D.L., Proweller A., Ishikawa M. (2014). Endothelial Kruppel-like factor 4 regulates angiogenesis and the Notch signaling pathway. J. Biol. Chem..

[150] Zhou G., Hamik A., Nayak L., Tian H., Shi H., Lu Y., Sharma N., Liao X., Hale A., Boerboom L. (2012). Endothelial Kruppel-like factor 4 protects against atherothrombosis in mice. J. Clin. Investig..

[151] Dambal S., Shah M., Mihelich B., Nonn L. (2015). The microRNA-183 cluster: The family that plays together stays together. Nucleic Acids Res..

